# Sub-5 nm lanthanide-doped lutetium oxyfluoride nanoprobes for ultrasensitive detection of prostate specific antigen†
†Electronic supplementary information (ESI) available: ESI, Tables S1–S4, Fig. S1–S20 and Movie S1. See DOI: 10.1039/c5sc04599a


**DOI:** 10.1039/c5sc04599a

**Published:** 2016-01-12

**Authors:** Jin Xu, Shanyong Zhou, Datao Tu, Wei Zheng, Ping Huang, Renfu Li, Zhuo Chen, Mingdong Huang, Xueyuan Chen

**Affiliations:** a Key Laboratory of Optoelectronic Materials Chemistry and Physics , Fujian Institute of Research on the Structure of Matter , Chinese Academy of Sciences , Fuzhou , Fujian 350002 , China . Email: xchen@fjirsm.ac.cn; b State Key Laboratory of Structural Chemistry , Danish-Chinese Centre for Proteases and Cancer , Fujian Institute of Research on the Structure of Matter , Chinese Academy of Sciences , Fuzhou , Fujian 350002 , China; c University of Chinese Academy of Sciences , Beijing 100049 , China

## Abstract

It remains challenging to develop ultrasmall (<5 nm) but highly luminescent bioprobes with a large linear detection range for the early diagnosis and monitoring of prostate cancer (PCa). Benefiting from the high molar density of lanthanide ions in an oxyfluoride matrix and the superior dissolution capability of Lu_6_O_5_F_8_ nanoparticles in the enhancer solution, we demonstrated the successful use of novel sub-5 nm Lu_6_O_5_F_8_:Eu^3+^ nanoprobes for the detection of prostate specific antigen (PSA) in clinical serum samples. The limit of detection for PSA is as low as 0.52 pg mL^–1^, which is almost a 200-fold improvement relative to that of a commercial dissociation-enhanced lanthanide fluoroimmunoassay (DELFIA) kit. The PSA levels detected in 23 patient serum samples were consistent with those measured independently by the DELFIA kit, showing the assay's reliability with a correlation coefficient of 97%. A linear range of 4 orders of magnitude ranging from 8.5 × 10^–4^ to 5.6 ng mL^–1^ for the assay of PSA was achieved, which is highly promising for the early diagnosis of PCa and monitoring of PCa relapse of patients after radical prostatectomy.

## Abstract 2

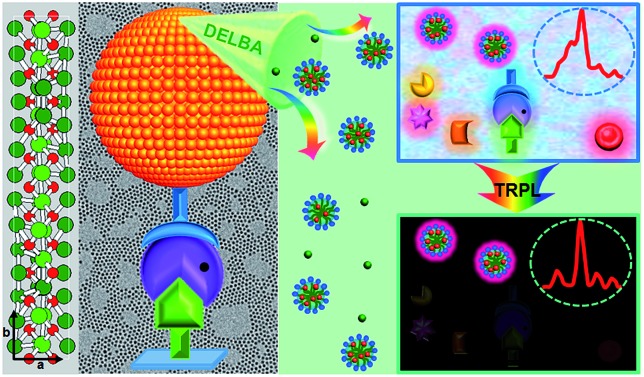
We demonstrate the successful use of sub-5 nm Lu_6_O_5_F_8_:Eu^3+^ nano-bioprobes for the ultrasensitive detection of prostate specific antigen in patient serum samples with a limit of detection of 0.52 pg mL^–1^.

## Introduction

Prostate cancer (PCa) is the third most common cancer in men worldwide.^1^ Prostate specific antigen (PSA) has been commonly used as a tumour marker for the early diagnosis and monitoring of PCa.^2^ For early diagnostic purposes, a PSA level of >4 ng mL^–1^ is considered as an indicator of suspicion of PCa. For the monitoring of PCa relapse, the PSA level in the serum usually decreases to ∼1 pg mL^–1^ after radical prostatectomy, and a serial increase in the PSA level indicates the recurrence or metastasis of PCa.^3^ Thus, ultrasensitive detection of PSA featuring a large linear range from ∼1 pg mL^–1^ to >4 ng mL^–1^ is crucial for the theranostics of PCa.

Tremendous efforts have been devoted to developing sensitive PSA-detecting approaches with a large linear range. Therein, fluorescence immunoassays are most commonly employed owing to their good compatibility with currently available analytical platforms in the clinic.^4^ However, commercial fluorescence bioassays have detection limits around 0.1 ng mL^–1^, which are insufficient for the monitoring of PCa relapse after radical prostatectomy. Therefore, an increased emphasis has been exerted on designing ultrasensitive assays with much better analytical sensitivity than commercial kits.^5^ Among fluorescence immunoassays previously established for the detection of PSA, time-resolved (TR) photoluminescence (PL) bioassays based on lanthanide-chelate embedded polystyrene or silica nanoparticles (NPs) are capable of achieving improved sensitivity than a commercial kit.^6^ Nevertheless, such nanoprobes (usually >40 nm) have a tendency to agglomerate and swell in aqueous solution, and potential chelate leakage concerns have also been documented.^6^,^7^ Compared with lanthanide chelates, lanthanide-doped inorganic NPs have become a research hotspot in biodetection owing to their relatively high stability and greater flexibility for bioconjugation.^8^–^18^ In particular, ultrasmall (<5 nm) nano-bioprobes in the size range of biological molecules are desired due to their minimized interference with the antigen–antibody binding process.^19^ Hitherto, it is still a challenge to develop ultrasmall lanthanide luminescent nano-bioprobes with both a high detection sensitivity and a large linear detection range. Recently, we have developed the dissolution-enhanced luminescent bioassay (DELBA) technique based on inorganic lanthanide fluoride NPs.^20^ Upon the addition of NPs to the enhancer solution (*i.e.* Triton X-100 micelle solution), the trivalent lanthanide (Ln^3+^) ions in the NPs are extracted into micelles containing chelating ligands to form highly luminescent lanthanide complexes. As such, the TRPL signals are amplified, improving the detection sensitivity. However, there is still great demand for exploring inorganic NPs with more highly concentrated Ln^3+^ ions and better dissolution performance to further improve the detection sensitivity and linear range of bioprobes. In comparison with fluorides, Ln^3+^ doped lutetium oxyfluorides, like Lu_6_O_5_F_8_, have a much higher molar density of Ln^3+^ ions in the matrix (ESI Table S1†). A larger amount of lanthanide luminescent micelles can be transformed from a single oxyfluoride NP. Besides, the Ln–O bond in Lu_6_O_5_F_8_ can be more easily loosened than the Ln–F bond *via* the protonation reaction with H^+^,^21^ which may facilitate Ln^3+^ ion extraction from the NP surface into micelles. As a result, Ln^3+^ doped Lu_6_O_5_F_8_ is expected to be more suitable than fluorides as an ultrasmall bioprobe for the detection of PSA.

**Fig. 1:**
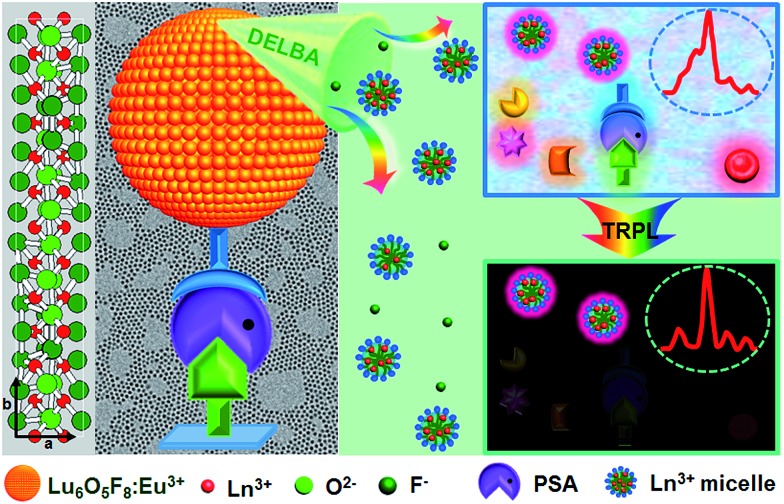
Schematic illustration of PSA detection utilizing sub-5 nm orthorhombic-phase Lu_6_O_5_F_8_:Eu^3+^ NPs as nanoprobes in TRPL mode.

Herein, monodisperse and ultrasmall Ln^3+^ (Ln = Eu, Yb/Er) doped lutetium oxyfluoride NPs, which had never been explored before, were synthesized *via* a modified thermal decomposition route.^22^ By utilizing the Lu_6_O_5_F_8_:Eu^3+^ nanoprobes featuring a high molar density of Ln^3+^ ions and superior dissolution properties in the enhancer solution, we demonstrate the ultrasensitive and accurate detection of PSA in patient serum samples through heterogeneous sandwich bioassays in the TRPL detection mode, as depicted in Fig. 1. Furthermore, we reveal the great potential of ultrasmall Lu_6_O_5_F_8_:Yb^3+^/Er^3+^ nanoprobes in upconversion luminescence (UCL) and computed tomography (CT) dual-modal bioimaging.

## Results and discussion

**Fig. 2:**
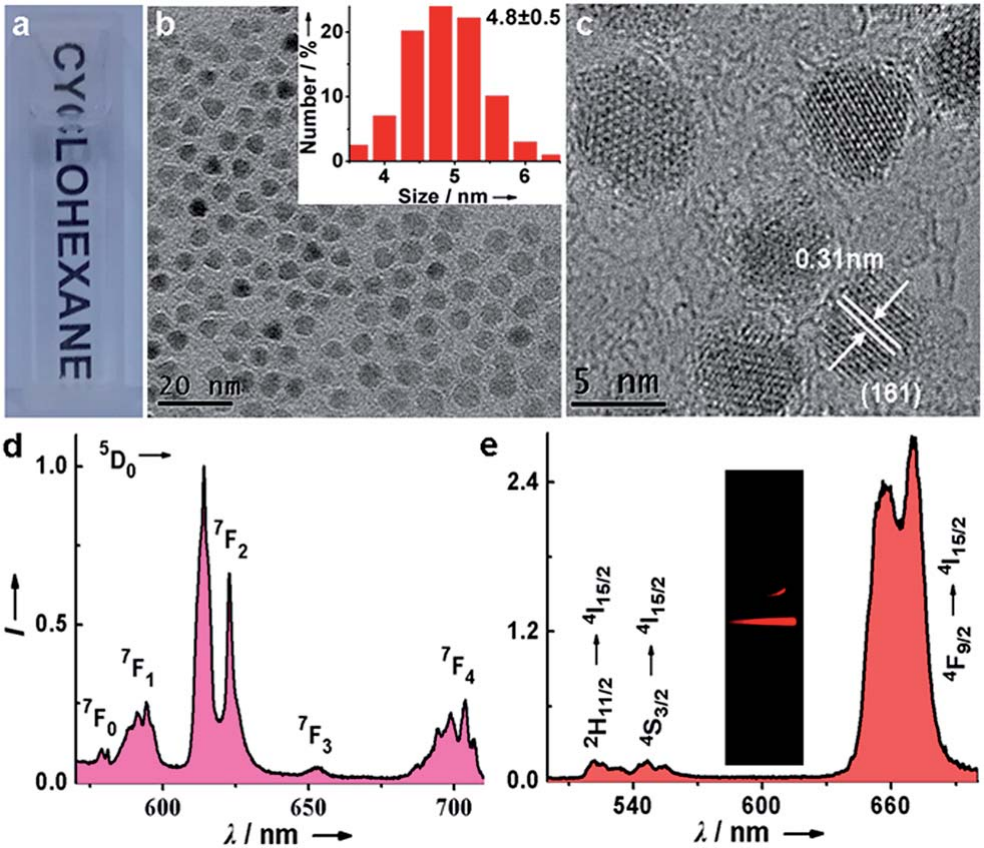
(a) Photograph showing the transparency of the as-prepared Lu_6_O_5_F_8_:Eu^3+^ (5 mol%) NPs dispersed in cyclohexane. (b) TEM and (c) HRTEM images of the as-prepared NPs. The inset shows a histogram of the size distribution. (d) PL emission spectrum for the Lu_6_O_5_F_8_:Eu^3+^ NPs upon excitation at 394 nm. (e) UCL spectrum of the as-prepared Lu_6_O_5_F_8_:Yb^3+^/Er^3+^ (10/2 mol%) NPs under 980 nm laser irradiation at a power density of ∼50 W cm^–2^. The inset shows the UCL photograph of the NPs dispersed in cyclohexane.

Monodisperse and ultrasmall Ln^3+^ (Ln = Eu, Yb/Er) doped lutetium oxyfluoride NPs were synthesized *via* a modified thermal decomposition route. The as-prepared Eu^3+^ (5 mol%) doped Lu_6_O_5_F_8_ NPs are readily dispersed in cyclohexane (Fig. 2a). TEM analysis shows that these NPs are nearly spherical with an average diameter of 4.8 ± 0.5 nm (Fig. 2b), as corroborated by the broad powder X-ray diffraction (XRD) peaks, which are well indexed as the orthorhombic phase of Lu_6_O_5_F_8_ (ESI Fig. S1†). The thermogravimetric analysis of the as-prepared NPs shows a weight loss of 5.17% between 450 and 750 °C (ESI Fig. S2†), which is consistent with the theoretical weight loss (5.19%) resulting from the decomposition of Lu_6_O_5_F_8_ to Lu_2_O_3_, thus verifying the chemical composition of the Lu_6_O_5_F_8_ NPs. The high-resolution TEM (HRTEM) image shows very clear lattice fringes with an observed *d*-spacing of 0.31 nm (Fig. 2c), which is in good agreement with the lattice spacing of the (161) plane of Lu_6_O_5_F_8_. Moreover, the sizes and morphologies of NPs with different Ln^3+^ doping levels remained essentially unchanged (ESI Fig. S3–S5†). Compositional analyses by energy-dispersive X-ray (EDX) spectroscopy and inductively coupled plasma-atomic emission spectroscopy (ICP-AES) revealed the existence of Lu, O, F, and the doping Ln^3+^ in the Lu_6_O_5_F_8_ NPs (ESI Fig. S6, Table S2†). Fig. 2d shows the characteristic Eu^3+^ downshifting (DS) emissions assigned to the ^5^D_0_ → ^7^F_0–4_ transitions in Lu_6_O_5_F_8_:Eu^3+^ (5 mol%) NPs upon excitation at 394 nm (^7^F_0_ → ^5^L_6_). The electric-dipole ^5^D_0_ → ^7^F_2_ transitions at ∼620 nm are much stronger than the magnetic-dipole ^5^D_0_ → ^7^F_1_ transitions at ∼590 nm, indicating that the doped Eu^3+^ ions occupy low-symmetry sites.^23^ The appearance of the ^5^D_0_ → ^7^F_0_ transitions infers that the site symmetry of Eu^3+^ in Lu_6_O_5_F_8_ should be restricted to noncentrosymmetric *C*_s_, *C*_*n*_, or *C*_*n*v_ (*n* = 1, 2, 3, 4, 6) groups,^23^ which is in accordance with the structural analysis that the doped Ln^3+^ ions may occupy several sites with symmetries of *C*_1_, *C*_s_ or *C*_2_ in Lu_6_O_5_F_8_.^24^ Meanwhile, in the corresponding excitation spectrum of the NPs (ESI Fig. S1b†), a broad O^2–^–Eu^3+^ charge transfer (CT) absorption in the 230–300 nm region was observed, which is typical of Eu^3+^ doped oxyfluoride hosts.^24^ Besides the intense DS luminescence, the colloidal cyclohexane solution of Lu_6_O_5_F_8_:Yb/Er (10/2 mol%) NPs displayed intense red UCL (inset of Fig. 2e). The UCL spectrum shows that the UCNPs exhibited strong red emission at ∼650 nm and relatively weak green emission at ∼540 nm with a red-to-green (R/G) ratio of ∼13, which are assigned to the ^4^F_9/2_ → ^4^I_15/2_ and ^2^H_11/2_/^4^S_3/2_ → ^4^I_15/2_ transitions of Er^3+^, respectively.^25^–^27^ Meanwhile, we observed a much shorter UCL lifetime of ^4^S_3/2_ (2.3 μs) than that of ^4^F_9/2_ (15.2 μs) of Er^3+^ (ESI Fig. S7†). The enhanced red UC emission may be attributed to the efficient energy back transfer (EBT) process from Er^3+^ to Yb^3+^ in the Lu_6_O_5_F_8_:Yb/Er lattice (ESI Fig. S8†).^28^,^29^ Such a high R/G ratio in UC phosphors is highly desired for *in vivo* bioimaging because the red UCL exhibits deeper tissue penetration than green light.^29^

**Fig. 3:**
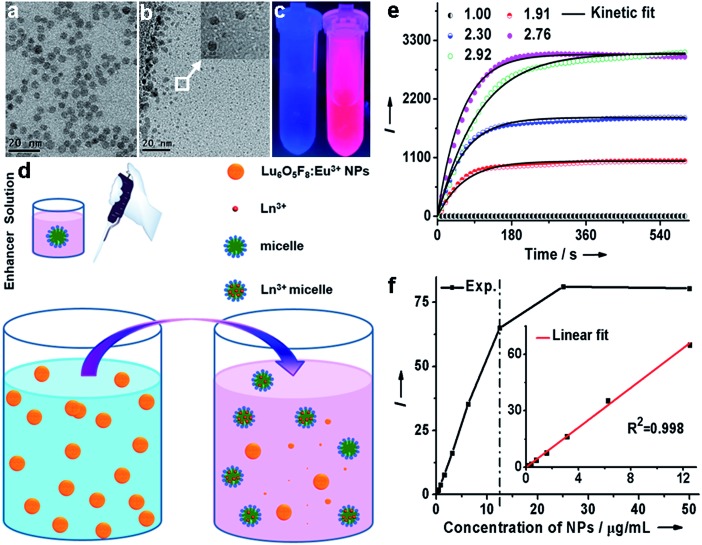
(a) Ligand-free Lu_6_O_5_F_8_:Eu^3+^ (40 mol%) NPs and (b) ligand-free NPs added to the enhancer solution. The inset shows an enlarged HRTEM image of the NPs, indicated by the white square. (c) Photograph showing the PL of ligand-free Lu_6_O_5_F_8_:Eu^3+^ NPs in a buffer solution (left) and the enhancer solution (right) under UV lamp illumination at 300 nm. (d) A schematic diagram for the mechanism of Lu_6_O_5_F_8_:Eu^3+^ NP dissolution. Upon addition of the enhancer solution to the solution of NPs, the massive Ln^3+^ ions accommodated in the NPs are extracted by the chelating ligands into micelles through spontaneous ligand–metal coordination reactions to form highly luminescent β-NTA–Ln^3+^–TOPO ternary complexes in the NP-micelle collisions. (e) Kinetic fit for the dissolution-enhanced PL signal of the ligand-free Lu_6_O_5_F_8_:Eu^3+^ (40 mol%) NPs (50 μg mL^–1^) dissolved in the enhancer solution at pH 1.00, 1.91, 2.30, 2.76 and 2.92. (f) Concentration-dependent dissolution-enhanced PL signal of ligand-free Lu_6_O_5_F_8_:Eu^3+^ NPs dissolved in the enhancer solution. Inset: the linear range of the PL signal *versus* the NP concentration (0–12.5 μg mL^–1^). (e–f) Each data point represents the mean of triplicate experiments.

To evaluate the dissolution performance of the Lu_6_O_5_F_8_:Eu^3+^ NPs, we firstly removed the oleate acid (OA) ligands from their surface by an acid-washing treatment to render the NPs hydrophilic.^30^ The successful removal of the surface ligands was confirmed by the Fourier transform infrared (FTIR) spectra (ESI Fig. S9†). Then, we added the solution of ligand-free Lu_6_O_5_F_8_:Eu^3+^ (40 mol%) NPs (20 μg mL^–1^) to the enhancer solution (*i.e.* the solution of Triton X-100 micelle containing 2-naphthoyltrifluoroacetone (β-NTA) and tri-*n*-octylphosphine oxide (TOPO) chelating ligands in the inner cavity, pH = 2.76). The appearance of a myriad of tiny NPs (<3 nm) in the TEM images indicates the occurrence of the dissolution reaction of the NPs, which was evidenced by the strong deep pink emission under ultraviolet (UV) lamp illumination upon addition of the NPs to the enhancer solution (Fig. 3a–c). To elucidate the origin of the dissolution-enhanced PL, we compared the steady-state PL excitation/emission spectra and the PL decays of the NPs before and after dissolution. The results show unambiguously that the enhanced Eu^3+^ PL of the NPs dissociated in the enhancer solution originated from the lanthanide complex, instead of the NPs (ESI Fig. S10†).

Since the lanthanide complexes are formed through the coordination reaction of the chelating ligands with the Ln^3+^ ions in the NPs (Fig. 3d), the dissolution-enhanced PL intensity of the Lu_6_O_5_F_8_:Eu^3+^ NPs depends critically on the molar concentration of Eu^3+^ in the NPs. Specifically, we found that Lu_6_O_5_F_8_ NPs doped with 40 mol% Eu^3+^ proved the most satisfactory (ESI Fig. S11†), due to the fact that the inert Lu^3+^ ion complex may decrease the concentration quenching of Eu^3+^ and enhance the luminescence of Eu^3+^ complex through a co-fluorescence effect.^31^ Similarly, the pH value of the enhancer solution has a complicated effect on the dissolution process of the NPs and the PL intensity of the Eu^3+^ complex (Fig. 3e).^31^ To gain deep insight into the dissolution dynamics of the NPs and to screen out optimal pH conditions for NP dissolution, we derived a kinetic equation (equation (S7) in ESI†) to simulate the dissolution process of the NPs. As shown in Fig. 3e and ESI Fig. S12,† the PL signal was well fitted by the kinetic equation. The kinetic parameters derived from the fitting are listed in ESI Table S3.† The results show that the equilibrium constant *K*_eq_ is the highest at pH 2.76. The higher value of *K*_eq_ indicates that a larger amount of NPs is dissolved when reaction equilibrium is reached. Consistently, the dissociation/association rate constants (*K*_d_/*K*_a_) are relatively high at pH 2.76, which means only a short time is needed to reach equilibrium in the dissolution reaction. As such, 2.76 was chosen as the optimal pH value for the dissolution of Lu_6_O_5_F_8_. Under these optimized conditions, we measured the linear dynamic range of dissolution-enhanced PL intensity *versus* the NP concentration. The PL signal increased linearly with the NP concentration in the large range of 0–12.5 μg mL^–1^ (*i.e.* 0–61.1 nmol Ln^3+^ per mL) (Fig. 3f). As shown in Fig. 3e, the dissolution-enhanced PL of NPs with a concentration even as high as 50 μg mL^–1^ was effectively stable within 5 minutes under optimum conditions. These results reveal the excellent dissolution performance of ultrasmall Lu_6_O_5_F_8_:Eu^3+^ nanoprobes for bioassays.

Prior to the luminescent bioassay, the OA-capped Lu_6_O_5_F_8_:Eu^3+^ NPs were surface-modified *via* ligand exchange with citrate, and then coupled with avidin following the EDC/NHS protocol.^32^,^33^ The successful conjugation of avidin to the surface of the NPs was corroborated by the appearance of amide bands in the FTIR spectrum, the increase in dynamic light scattering (DLS) size and the decrease of the zeta-potential for the NPs after surface modification (ESI Fig. S13 and S14†). By utilizing the bicinchoninic acid (BCA) protein assay kit, the number of avidin molecules conjugated to each NP was estimated to be ∼1.8 (ESI Fig. S15 and S16†).

**Fig. 4:**
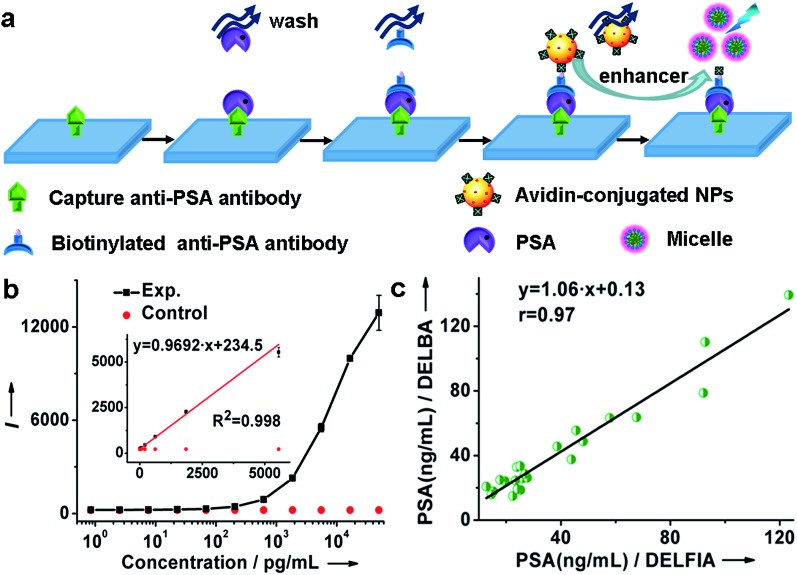
(a) The process and principle of a heterogeneous assay for the detection of PSA. (b) Calibration curve for the PSA assay based on Lu_6_O_5_F_8_:Eu^3+^ (40 mol%) NPs. Inset: the linear range (8.5 × 10^–4^ to 5.6 ng mL^–1^) of the calibration curve with the regression equation of *y* = 0.9692*x* + 234.5 (*R*^2^ = 0.998). (c) Correlation between the NP-based assay and commercial DELFIA kit for the detection of PSA in 23 patient serum samples. (b and c) Each data point represents the mean (±standard deviation) of triplicate experiments.

By virtue of the specific recognition of the anti-PSA antibody with PSA, we employed avidin-conjugated Lu_6_O_5_F_8_:Eu^3+^ nanoprobes in a sandwich-type bioassay for the detection of PSA. As illustrated in Fig. 4a and ESI Movie S1,† the capture antibody was first bound to the microplate well, and the biotinylated detection antibody (which was bound to PSA) was coupled to the nanoprobe through biotin–avidin interactions. PSA was quantified by measuring the dissolution-enhanced TRPL signal of the oxyfluoride nanoprobes upon addition of the enhancer solution on a microplate reader. For comparison, control experiments by replacing PSA with bovine serum albumin (BSA) under otherwise identical conditions, were also conducted. These control experiments showed negligible TRPL signal (Fig. 4b), verifying the high specificity. The limit of detection (LOD), defined as the concentration that corresponds to three times the standard deviation above the signal measured in the control experiment, was determined to be 0.52 pg mL^–1^ (15.2 fM). The derived LOD exhibits an almost 200-fold improvement relative to that of a commercial dissociation-enhanced lanthanide fluoroimmunoassay (DELFIA) kit (0.1 ng mL^–1^),^34^ and is the lowest among lanthanide nanoprobes ever reported for the luminescent bioassay of PSA (ESI Table S4†). Such a low LOD is attributed to the high molar density of Eu^3+^ ions in a single oxyfluoride NP and the high specificity of nanoprobes in the bioassays.

It is worth emphasizing that the luminescent bioassay based on ultrasmall oxyfluoride nanoprobes demonstrates an excellent linear dependence of the PL intensity on PSA concentration over a rather wide range, namely, 8.5 × 10^–4^ to 5.6 ng mL^–1^ (*R*^2^ = 0.998) (Fig. 4b and ESI Fig. S17†). According to Diamandis *et al.*, the PSA levels in ∼60% of patients suffering from PCa decreased to <5 pg mL^–1^ after radical prostatectomy,^35^ and a postoperative increase of serum PSA indicates the recurrence or metastasis of cancer.^3^ Benefiting from the ultrahigh sensitivity of our nanoprobe, such a large linear dynamic range spanning four orders of magnitude for a PSA bioassay might fulfil the critical requirement for tracing the serum PSA level for the early diagnosis of PCa and for monitoring PCa relapse after prostatectomy.

To further verify the applicability of the Lu_6_O_5_F_8_:Eu^3+^ nanoprobes in practical bioassays for PSA, we firstly studied the response of the oxyfluoride probes towards potential interfering proteins coexisting in serum samples. PSA (300 pM) and noncognate proteins (10 nM), including human serum albumin (HSA), carcinoembryonic antigen (CEA), alpha fetoprotein (AFP), and beta-human chorionic gonadotropin (β-HCG), were added to prepared serum samples from a healthy male with an original PSA level of 0.54 ng mL^–1^ (15.88 pM) determined by a commercial DELFIA kit, respectively. In the blank control, no targets were added to the samples. As shown in ESI Fig. S18,† the PL signals of all the samples with noncognate proteins added were indistinguishable from that of the blank control. By contrast, when the standard solution of PSA was added, even with its concentration more than 30-fold lower than that of the noncognate protein, a greater than 7 times stronger PL signal was observed. These results indicate that nonspecific binding is negligible, thus confirming the great specificity of our proposed method for the detection of PSA in complex human serum samples. Then, we carried out *in vitro* detection of PSA in 23 serum samples of patients with PSA levels from 12.00 to 140.00 ng mL^–1^. The PSA levels determined were compared with those independently detected using a commercial DELFIA kit. As shown in Fig. 4c and Table 1, the PSA levels determined from the DELBA assay based on Lu_6_O_5_F_8_:Eu^3+^ NPs are consistent with those from the DELFIA. The correlation coefficient between both kinds of assays was determined to be 0.97, indicating that the NPs-based assay results are as reliable as that of the commercial DELFIA. The coefficients of variation (CV) of the Lu_6_O_5_F_8_:Eu^3+^-based assay ranged from 0.2% to 8.5% (Table 1), showing the assay's good reproducibility. Moreover, we evaluated the analytical accuracy and precision of the Lu_6_O_5_F_8_:Eu^3+^ bioprobe through the determination of PSA levels, CV, and the recovery of two serum samples from healthy males upon the addition of PSA standard solutions with different concentrations. The CVs of all the assays are below 8% and the analytical recoveries are in the range of 92–108% (Table 2), both of which are within the acceptance criteria (CVs ≤ 15%; recoveries in the range of 90–110%) set for bioanalytical method validation.^20^ These results verify that the Lu_6_O_5_F_8_:Eu^3+^ bioprobe has high reliability and practicability for PSA detection.

**Table 1:** Comparison of the PSA levels in 23 patient serum samples determined by assays based on Lu_6_O_5_F_8_:Eu^3+^ NPs and commercial DELFIA (mean ± standard deviation (SD), coefficient of variation (CV), *n* = 3)

No.	NPs-based assay		DELFIA	No.	NPs-based assay		DELFIA
	Mean ± SD (ng mL^–1^)	CV%	Mean ± SD (ng mL^–1^)		Mean ± SD (ng mL^–1^)	CV%	Mean ± SD (ng mL^–1^)
1	25.84 ± 1.12	4.3	26.03 ± 0.50	13	37.61 ± 0.83	2.2	43.88 ± 0.38
2	24.06 ± 0.22	0.9	19.60 ± 0.43	14	45.60 ± 0.98	2.1	38.69 ± 0.60
3	17.75 ± 0.39	2.2	15.36 ± 0.27	15	110.32 ± 0.63	0.6	92.76 ± 0.70
4	63.20 ± 1.77	2.8	57.86 ± 1.01	16	55.49 ± 1.31	2.4	45.56 ± 0.67
5	20.72 ± 0.94	4.5	12.78 ± 0.65	17	32.83 ± 1.85	5.6	23.86 ± 0.44
6	139.39 ± 1.89	1.4	123.32 ± 0.79	18	24.86 ± 1.21	4.9	17.74 ± 0.16
7	63.64 ± 0.68	1.1	67.65 ± 0.62	19	15.92 ± 1.35	8.5	14.66 ± 0.31
8	28.58 ± 0.54	1.9	26.93 ± 0.61	20	19.20 ± 0.53	2.8	24.12 ± 0.29
9	78.67 ± 1.16	1.5	92.09 ± 0.96	21	26.22 ± 0.06	0.2	27.74 ± 0.35
10	18.64 ± 1.48	7.9	25.26 ± 0.25	22	33.42 ± 0.08	0.2	25.15 ± 0.56
11	14.82 ± 0.40	2.7	22.44 ± 0.48	23	24.50 ± 1.21	4.9	23.40 ± 0.32
12	48.73 ± 0.54	1.1	48.20 ± 0.82				

**Table 2:** Assay precision and analytical recovery of PSA added to two serum samples from healthy males

Added (ng mL^–1^)	Found (ng mL^–1^)	CV (%) *n* = 4	Recovery (%)
Sample 1	2.99	3.57	—
2.50	5.65	3.98	106.51
3.00	5.78	2.13	93.24
12.00	15.87	1.06	107.36
Sample 2	1.83	5.09	—
1.00	2.81	7.36	98.17
7.00	9.01	2.88	102.59
28.00	27.76	2.03	92.60

To demonstrate the multifunctionality of the oxyfluoride nanoprobes, we also employed ultrasmall Lu_6_O_5_F_8_:Yb/Er NPs in proof-of-concept UCL and CT bioimaging, in view of the high R/G UC emissions and the high mass density of the Lu_6_O_5_F_8_:Yb/Er NPs (∼9 g cm^–3^). After incubation with human lung cancer (H1299) cells, strong red UC emissions of Er^3+^ were clearly visualized in the cells upon 980 nm irradiation, in sharp contrast to the weak green luminescence observed in the dark (ESI Fig. S19a†), which agrees well with the corresponding nearly single-band UC spectrum (Fig. 2e). In addition, as a proof-of-concept experiment, we recorded *in vitro* color-mapped CT images and measured the CT values using an aqueous solution of Lu_6_O_5_F_8_:Yb/Er NPs. The CT value of NPs at 12.5 mg mL^–1^ was 319 Hounsfield units (HU), which is much higher than that of LiLuF_4_ NPs (181 HU) and commercial iopromide (186 HU) at the same concentration (ESI Fig. S19b and c†).^36^ To further evaluate the cytotoxicity of the citrate-capped Lu_6_O_5_F_8_:Yb/Er NPs for potential bioimaging applications, the dark/photo toxicity of the NPs were measured against HELF cells using a standard methylthiazolyltetrazolium (MTT) assay. There is no significant cytotoxicity of the NPs (ESI Fig. S20†), even at a high concentration of 1 mg mL^–1^, either in the dark or upon 980 nm irradiation for 2 min. These results demonstrate the potential of the UC NPs as bioprobes for UCL/CT dual-modal bioimaging.

## Conclusions

In summary, we have developed sub-5 nm Lu_6_O_5_F_8_:Eu^3+^ NPs as the first inorganic oxyfluoride nanoprobe for the detection of PSA. Because of the high molar density of Ln^3+^ ions in the Lu_6_O_5_F_8_ NPs, with superior dissolution capability in the enhancer solution and the minimized non-specific binding of the NPs in TRPL bioassays, we have achieved an exceptionally large linear dynamic range spanning from 0.85 pg mL^–1^ to 5.6 ng mL^–1^, along with a LOD as low as to 0.52 pg mL^–1^, which is more than two orders of magnitude lower than that of a commercial DELFIA kit. More importantly, we have successfully employed the ultrasmall nanoprobes for the detection of PSA in 23 clinical serum samples of patients suffering from PCa, and the assay results were highly consistent with those measured independently by DELFIA kit, showing the assay's reliability with a correlation coefficient of 0.97. These findings demonstrate the great potential of such a nano-bioprobe in the practical *in vitro* detection of tumor markers, and particularly, for monitoring PCa relapse of patients after radical prostatectomy, which may eventually open up a new route to the exploitation of oxyfluoride nanoprobes in versatile biomedical applications.

## Supplementary Material

Supplementary movie

Supplementary information
