# Supplementary material for: Sub-5 nm lanthanide-doped lutetium oxyfluoride nanoprobes for ultrasensitive detection of prostate specific antigen
Source: Chem Sci. 2016 Jan 12;7(4):2572–8. doi: 10.1039/c5sc04599a (PMC5477113; doi:10.1039/c5sc04599a)
Supplement: Supplementary file 2 [file SC-007-C5SC04599A-s002.pdf]

Electronic Supplementary Material (ESI) for Chemical Science.
This journal is © The Royal Society of Chemistry 2016

Electronic Supplementary Information (ESI) for Chemical Science

Sub-5 nm Lanthanide-Doped Lutetium Oxyfluoride Nanoprobes for Ultrasensitive Detection of Prostate Specific Antigen

Jin Xu,[a,b,c] Shanyong Zhou,[a] Datao Tu,[a] Wei Zheng,[a] Ping Huang,[a] Renfu Li,[a] Zhuo Chen,[b] Mingdong Huang,[b] and Xueyuan Chen*[a,b,c]

[a.]Key Laboratory of Optoelectronic Materials Chemistry and Physics, Fujian Institute of Research on the Structure of Matter, Chinese Academy of Sciences, Fuzhou, Fujian 350002, China

[b.]State Key Laboratory of Structural Chemistry, and Danish-Chinese Centre for Proteases and Cancer, Fujian Institute of Research on the Structure of Matter, Chinese Academy of Sciences, Fuzhou, Fujian 350002, China

[c.]University of Chinese Academy of Sciences, Beijing 100049 (China)

*To whom correspondence should be addressed, e-mail: xchen@fjirsm.ac.cn

I. Supplementary Materials & Methods

Chemicals and Materials: Ln_2O_3 (Ln = Lu, Eu, Yb, Er) (99.99%), trifluoroacetic acid, 2-hydroxy-1,2,3,-propanetricarboxylic acid trisodium salt (analytical grade), acetic acid sodium salt trihydrate (analytical grade), glacial acetic acid (analytical grade), hydrochloric acid (analytical grade), cyclohexane, ethanol, chloroform and dimethyl sulfoxide (DMSO) were purchased from Sinopharm Chemical Reagent Co., China. Oleic acid (OA), oleylamine (OM), 1-ethyl-3-(3-dimethyllaminopropyl)-carbodiimide hydrochloride (EDC), *N*-hydroxysuccinimide (NHS), 2-(N-morpholino)ethanesulfonic acid (MES), 2-naphthoyltrifluoroacetone (β-NTA), tri-n-octylphosphine oxide (TOPO), Triton X-100, biotin, avidin, bovine serum albumin (BSA) and human serum albumin (HSA) were purchased from Sigma-Aldrich (China). Bicinchoninic acid (BCA) protein assay kit, the standard solutions of prostate specific antigen (PSA), mouse anti-PSA monoclonal antibody, biotinylated mouse anti-PSA monoclonal antibody, carcinoembryonic antigen (CEA), alpha fetoprotein (AFP), and beta-human chorionic gonadotropin (β-HCG) were purchased from Shanghai Linc-Bio Science Co. Dissociation-enhanced lanthanide fluoroimmunoassay (DELFIA) kit for PSA was purchased from Daan Gene Co., Ltd. of Sun Yat-Sen University, China. Human serum samples were kindly provided by Fujian Provincial Cancer Hospital, Fuzhou, China. The 96-well Nunc Immobilizer Amino plate was purchased from Thermo Fisher Scientific Inc., which has a high affinity for the coupling of peptide and protein in fluoroimmunoassays. All the chemical reagents were used as received without further purification.

Synthesis of $Lu_6O_5F_8:Ln^{3+}$ Nanoparticles (NPs): Monodisperse $Lu_6O_5F_8:Ln^{3+}$ NPs (Ln = Eu, Yb/Er) were synthesized *via* a modified thermal decomposition process.[1] $Ln(CF_3COO)_3$ was prepared as reported in the literature.[2] In a typical process of synthesizing $Lu_6O_5F_8:Eu^{3+}$ (5 mol%) NPs, 0.95 mmol $Lu(CF_3COO)_3$ and 0.05 mmol $Eu(CF_3COO)_3$ were mixed with 6 mL of OA and 8 mL of OM in a 100 mL three-neck round-bottom flask. The obtained mixture was heated at 160 ℃ for 30 min under constant magnetic stirring in N_2 atmosphere, in order to dissolve trifluoroacetate and simultaneously to remove residual water and oxygen. Subsequently, the resulting transparent solution was heated to 320 ℃ under N_2 flow with vigorous stirring for 120 min, and then cooled to room temperature (RT) naturally. The resulting $Lu_6O_5F_8:Eu^{3+}$ (5 mol%) NPs were precipitated by addition of ethanol, collected *via* centrifugation, washed with ethanol for several times, and re-dispersed in cyclohexane.

Synthesis of Ligand-Free $Lu_6O_5F_8:Ln^{3+}$ Nanoparticles: Ligand-free $Lu_6O_5F_8:Ln^{3+}$ NPs were obtained by removing the surface ligands of oleate-capped counterparts through acid-washing treatment.[3] Typically, 60 mg of as-prepared oleate-capped $Lu_6O_5F_8:Ln^{3+}$ NPs were dispersed in 15 mL of acidic ethanol solution (pH 1; prepared by adding 112 μL of concentrated hydrochloric acid to 15 mL of absolute ethanol) and ultrasonicated for 30 min to remove the surface ligands. After the reaction, the

NPs were collected by centrifugation at 13600 rpm for 30 min. The resulting products were washed with ethanol and distilled water several times, and then re-dispersed in distilled water.

Synthesis of Citrate-Capped $Lu_6O_5F_8:Ln^{3+}$ NPs: Citrate modification of $Lu_6O_5F_8:Ln^{3+}$ NPs were performed *via* a modified ligand exchange process.[4] Briefly, the as-prepared oleate-capped NPs (30 mg) were dispersed in a mixture solution containing 2 mL of chloroform, 2 mL of ethanol and 2 mL of sodium citrate solution (0.2 M). The resulting mixture solution was then stirred for 12 h at RT. Thereafter, the mixture solution was allowed to delaminate until a sharp demarcation line emerged between water and chloroform layers. Afterwards, the upper colloidal aqueous phase was collected and centrifugated at 13600 rpm for 30 min to yield citrate-capped NPs. The resulting products were washed with ethanol and distilled water several times, and then re-dispersed in distilled water.

Synthesis of Avidin-Conjugated $Lu_6O_5F_8:Ln^{3+}$ NPs: Citrate-capped $Lu_6O_5F_8:Ln^{3+}$ NPs was conjugated with avidin following the well-established EDC/NHS protocol.[5] 1 mL of citrate-capped $Lu_6O_5F_8:Ln^{3+}$ NPs (1 mg/mL in 10 mM MES buffer) were activated with 20 mM EDC and 50 mM NHS for about 3 h at RT. Then, the activated NPs were purified by centrifuging at 13600 rpm for 30 min and incubated with 1 mL of avidin (1 mg/mL) in phosphate buffered saline (PBS, pH 7.2). After overnight shaking at 4 ℃, the unbound avidin was removed from avidin-conjugated $Lu_6O_5F_8:Ln^{3+}$ NPs by centrifugation at 13600 rpm for 30 min. The residual NPs were washed several times with distilled water. Finally, avidin-conjugated $Lu_6O_5F_8:Ln^{3+}$ NPs were re-dispersed in distilled water and stored at 4 ℃.

Quantitative Analysis of Avidin on the Surface of $Lu_6O_5F_8:Ln^{3+}$ NPs: The amount of avidin coupled to citrate-caped $Lu_6O_5F_8:Ln^{3+}$ NPs can be approximately quantified on the basis of a standard BCA protein assay protocol.[6] A standard curve was prepared as described in the protocol. The avidin content on the surface of citrate-caped $Lu_6O_5F_8:Ln^{3+}$ NPs was determined from the standard curve with BSA as a reference.

Preparation of the enhancer solution: We prepared the enhancer solutions at different pH values with a modified formulation as previously reported.[7] Firstly, buffered 0.1% (m/v) Triton X-100 solutions were prepared by adjusting sodium acetate buffer (100 mM) to the desired pH with glacial acetic acid and hydrochloric acid if necessary. All pH measurements were done at RT using a Sartorius PB-10 pH meter (Sartorius, Germany). Different pH values of 1.00, 1.91, 2.30, 2.76, 2.92, 3.60 and 5.50 were chosen. Then, a second solution prepared by dissolving appropriate concentrations of β-NTA and TOPO in a suitable volume of ethanol was added to the buffered Triton X-100 solutions under stirring,

respectively. Finally, the resulting solutions containing 15 μM β-NTA and 50 μM TOPO at different pH values were stored at 4 ℃.

Detection response and linear dynamic range of ligand-free $Lu_6O_5F_8:Ln^{3+}$ NPs: Time-dependent dissolution-enhanced photoluminescence (PL) signals of ligand-free $Lu_6O_5F_8:Eu^{3+}$ NPs in buffer solution (50 μg/mL, 100 μL) at pH 1.00, 1.91, 2.30, 2.76, 2.92, 3.60 and 5.50 were measured at RT under the kinetic and time-resolved (TR) detection mode on a multimodal microplate reader (Synergy 4, BioTek) immediately upon addition of 100 μL of the enhancer solution at the same pH value as the corresponding NP solution, respectively. The total measuring time and time intervals for the signal collection were set to be 600 and 10 s, respectively. Concentration-dependent dissolution-enhanced PL signal of ligand-free $Lu_6O_5F_8:Eu^{3+}$ NPs (0-50 μg/mL in buffer solution at pH 2.76, 100 μL) was recorded under the TR detection mode on the multimodal microplate reader at RT after 10 min upon addition of the enhancer solution (pH 2.76, 100 μL). The excitation and emission wavelengths were 340 nm and 614 nm, respectively. The delay time and gate time were set to be 200 μs and 2 ms, respectively. Three independent experiments were carried out to yield the mean value and deviation.

PSA assay based on dissolution-enhanced PL of $Lu_6O_5F_8:Eu^{3+}$ nanoprobes: After anti-PSA monoclonal antibody (diluted to 10 μg/mL with 100 mM carbonate buffer of pH 9.6) was coated on the wells (100 μL per well) of a 96-well Nunc Immobilizer Amino plate *via* incubation at 37 ℃ for 1 h, 300 μL of blocking solution (100 mM carbonate buffer containing 0.1% of 2-aminoethanol, pH 9.6) was added in each well and incubated at 37 ℃ for another 1 h to block other free-standing groups on the plate. The plate was washed with PBST (Phosphate Buffered Saline containing 150 mM of NaCl and 0.05% (v/v) TWEEN 20, pH 7.2) for 3 times. PSA standard solution (100 μL per well) was then added to each well. After incubation at 37 ℃ for 1 h, the wells were washed with PBST for 3 times. Then biotinylated anti-PSA monoclonal antibody (2 μg/mL, 100 μL per well) was added to each well and the plate was incubated at 37 ℃ for 1 h. Subsequently, the plate was aspirated and washed with PBST for 3 times. Thereafter, avidin-conjugated $Lu_6O_5F_8:Eu^{3+}$ (40 mol%) NPs (20 μg/mL, 100 μL per well) was added to each well and the plate was incubated at 37 ℃ for 1 h. After washing with PBST for 6 times, the enhancer solution (200 μL) was added into each well, and then the plate was subjected to TRPL detection on a multimodal microplate reader (Synergy 4, BioTeK) at RT. The excitation and emission wavelengths were 340 nm and 614 nm, respectively. The delay time and gate time were set to be 200 μs and 2 ms, respectively. For comparison, control experiments by replacing PSA with BSA under otherwise identical conditions were also conducted. The assay of human serum samples was conducted following the same procedure by simply replacing the PSA standard solution with human serum samples (each plate well was added with 20 μL of serum and 80 μL of PBS). The PSA levels in human

serum samples (diluted 1:5 in PBS) were determined using the regression equation derived from the regression analysis of the calibration curve generated by the assay of PSA standard solution (Figure S17). Three independent experiments were carried out to yield the mean value and deviation.

PSA assay based on commercial DELFIA kit: The PSA levels in human serum samples can be approximately quantified following a protocol of commercial DELFIA kit for PSA (Daan Gene Co., Ltd. of Sun Yat-Sen University, China). A calibration curve was prepared as described in the protocol. The PSA levels in human serum samples were then determined from the calibration curve.

Dark Cytotoxicity and Phototoxicity of Citrate-Capped $Lu_6O_5F_8$:Yb/Er NPs: Human embryo lung fibroblast (HELF) cells at a density of 2×10^4 cells per milliliter were placed in 96-multiwell plates with a volume of 200 μL per well and incubated overnight at 37 °C under 5% CO_2. The cells were incubated with different concentrations of citrate-capped $Lu_6O_5F_8$:Yb/Er NPs (0, 31.25, 62.5, 125, 250, 500 and 1000 μg/mL) for a period of 12 h. One control column in the plate was filled with fresh culture medium only. The cells were then washed twice with sterile PBS before fresh medium was added. Methylthiazolyltetrazolium (MTT) was subsequently applied to the cells followed by incubation at 37 ℃ under 5% CO_2 for 4 h, and then the culture medium was carefully removed and replaced with DMSO. The OD_{490} value of each well was measured on a multimodal microplate reader (Synergy 4, BioTek). The following formula was applied to calculate the percent inhibition rate of cell growth: cell viability (%) = (mean of absorbance value of treatment group / mean of absorbance value of control) × 100%. Four replicates were run per NPs dose in each cell lines. The same protocol was utilized to determine the phototoxicity of citrate-capped $Lu_6O_5F_8$:Yb/Er NPs to HELF cells, except that the HELF cells were irradiated by using a 980 nm laser for 2 min after incubation of citrate-capped $Lu_6O_5F_8$:Yb/Er NPs with HELF cells for 12 h.

Cell Culture and Confocal Laser Scanning Microscopy: Human lung cancer (H1299) cell line was purchased from Shanghai Institute of Cell Biology, Chinese Academy of Sciences, and was routinely maintained in RPMI-1640 (GIBCO BRL), supplemented with 10% (v/v) heat-inactivated fetal calf serum, penicillin (100 U/mL), and streptomycin (100 U/mL) at 37 ℃ under humidified air containing 5% CO_2. H1299 cells were seeded into culture plates and allowed to adhere for 24 h. After washing with PBS (pH 7.2) several times, the cells were incubated in culture medium (RPMI-1640) containing 0.5 mg/mL of citrate-capped $Lu_6O_5F_8:Yb^{3+}/Er^{3+}$ NPs at 37 ℃ for 2 h under 5% CO_2, and then washed with PBS sufficiently to remove the excess NPs. The cell imaging was performed by using confocal laser scanning microscope equipped with an Olympus FV1000 scanning unit. Cells were excited by a

980 nm laser and upconversion luminescence (UCL) signals were collected in the green channel (520-560 nm) and red channel (640-680 nm), respectively.

X-ray Attenuation Measurement for Computed Tomography (CT) Imaging: Different concentrations of citrate-capped $Lu_6O_5F_8$:Yb/Er NPs (25, 12.5, 6.25 and 3.125 mg/mL) dispersed in distilled water were prepared in a series of 1.5 mL centrifugation tubes as contrast agents for CT test. CT images were acquired using a dual-source CT system (SIEMENS Inveon MMCT micro CT instrument). Imaging parameters were as follows: effective pixel size, 105.61 μm; 80 KVp, 500 μA; field of view, 54.07 mm×146.00 mm; rotation steps, 180; binning, 4; exposure time 150 ms/rotation. Phantom and color-mapped CT images were analyzed with Kodak Molecular Imaging Software. Hounsfield units (HU) values were measured by the SIEMENS Inveon MMCT micro CT software.

Structural and Optical Characterization: Powder X-ray diffraction (XRD) patterns of the samples were collected on an X-ray diffractometer (MiniFlex2, Rigaku) with Cu Kα1 radiation (λ = 0.154187 nm). Transmission electron microscopy (TEM) measurements were performed on a JEOL-2010 TEM equipped with the energy-dispersive X-ray (EDX) spectrum. The quantitative analysis of element content in NPs was performed with inductively coupled plasma-atomic emission spectroscopy (ICP-AES, Ultima2, Jobin Yvon). Thermogravimetric analysis (TGA) was conducted on a Netzsch STA449C thermal analysis system under air atmosphere flow at a rate of 10 ℃/min. Fourier transform infrared (FTIR) spectra were recorded in KBr discs on a Magna 750 FTIR spectrometer. The hydrodynamic diameter distribution and ζ-potential of the synthesized $Lu_6O_5F_8$:Ln^{3+} NPs dispersed in distilled water were determined by means of dynamic light scattering (DLS) measurement (Nano ZS ZEN3600, Malvern).

Downshifting (DS) PL excitation / emission spectra and PL decays were recorded on a spectrometer equipped with both continuous (450 W) xenon and pulsed flash lamps (FLS920, Edinburgh Instrument). UC emission spectra was collected under 980-nm laser excitation at ~50 W/cm^2 provided by a continuous-wave laser diode. UCL lifetimes were measured with a customized ultraviolet (UV) to mid-infrared steady-state and phosphorescence lifetime spectrometer (FSP920-C, Edinburgh) equipped with a digital oscilloscope (TDS3052B, Tektronix) and a tunable mid-band Optical Parametric Oscillator (OPO) pulse laser as the excitation source (410-2400 nm, 10 Hz, pulse width of ~5 ns, Vibrant 355II, OPOTEK). All the spectral data collected were corrected for the spectral response of the spectrometer. PL photographs of the NP solutions were taken by a Sony H20 digital camera without using any filter.

Dissolution of $Lu_6O_5F_8$:Eu^{3+} NPs in the Enhancer Solution: The dissolution of $Lu_6O_5F_8$:Eu^{3+} NPs (for simplicity, abbreviated as F-Ln-O) is described by the following reactions:

(a) When the ligand-free NPs are suspended in a weakly acidic enhancer solution, the surfaces of NPs behave as a Lewis base, which is described by the following protonation processes of Ln-O/F bonds:

$$\text{F-Ln-O} + 2\text{H}^+ \rightleftharpoons \text{H}^+\text{...F-Ln-O...H}^+ \qquad \text{(a)}$$

According to the above reaction, the NP surfaces become positively charged, and simultaneously the Ln-O/F bonds are loosened due to the protonation process.

(b) To account for the hypothesis that the Ln^{3+} ions accommodated in NPs are continuously extracted from the NP surfaces into Triton X-100 micelles containing β-NTA and TOPO ligands through the spontaneous ligand-metal coordination reactions to form highly luminescent lanthanide complexes in the NP-micelle collisions, the dissolution reaction may be written as

$$\text{H}^+\text{...F-Ln-O...H}^+ + \left\langle \text{HNTA}_3 \cdot \text{TOPO}_{2,3} \right\rangle(\text{micelle}) \rightleftharpoons \text{Ln(NTA)}_3(\text{TOPO})_{2,3}(\text{micelle}) + \text{H}_2\text{O} + 2\text{H}^+ + \text{HF} \quad \text{(b)}$$

where $\langle HNTA_3 \cdot TOPO_{2,3} \rangle$ represents a coordination unit of the mixed β-NTA and TOPO ligands in micelle. According to reaction (b), the extraction processes are directly affected by the protonation reactions of Ln-O/F bonds with H^+ and the deprotonation processes of chelating ligand in coordination reactions with Ln^{3+} ions.

(c) The total dissolution reaction of $Lu_6O_5F_8:Eu^{3+}$ NPs = reaction (a) + reaction (b):

$$\text{F-Ln-O} + \left\langle \text{HNTA}_3 \cdot \text{TOPO}_{2,3} \right\rangle(\text{micelle}) \rightleftharpoons \text{Ln(NTA)}_3(\text{TOPO})_{2,3}(\text{micelle}) + \text{H}_2\text{O} + \text{HF} \qquad \text{(c)}$$

Kinetic Analysis of $Lu_6O_5F_8:Eu^{3+}$ NPs Dissolution: The dissolution reaction of $Lu_6O_5F_8:Eu^{3+}$ NPs in the enhancer solution and the total concentrations of each species (C) at time t may be written as

$$\begin{array}{llll} \text{F-Ln-O} + & \left\langle \text{HNTA}_3 \cdot \text{TOPO}_{2,3} \right\rangle(\text{micelle}) & \underset{K_a'}{\overset{K_d'}{\rightleftharpoons}} \text{Ln(NTA)}_3(\text{TOPO})_{2,3}(\text{micelle}) + \text{H}_2\text{O} + & \text{HF} \\ C(t):\ a'-x' & c'-x' & x' & x' \end{array}$$

where a' and c' are the initial total molar concentrations of F-Ln-O unit in NPs, and coordination unit of β-NTA and TOPO ligands in Triton X-100 micelles, respectively; x' is the amount of F-Ln^{3+}-O unit dissolved at time t; K_d' and K_a' are the dissociation rate constant and association rate constant, respectively. Assuming both the dissociation / association reactions are first order with respect to the total concentration of each species, the kinetic equation can be expressed as

$$\frac{dx'}{dt} = K_d'\left(a'-x'\right)\left(c'-x'\right) - K_a' x'^2 \qquad \text{(S1)}$$

Assuming a_0 is the initial total molar concentration of F-Ln-O unit in 50 μg/mL ligand-free NPs, the kinetic equation can be written as

$$\frac{dx'/a_0}{dt} = K_d{}'a_0\left(\frac{a'}{a_0}-\frac{x'}{a_0}\right)\left(\frac{c'}{a_0}-\frac{x'}{a_0}\right)-K_a{}'a_0\left(\frac{x'}{a_0}\right)^2 \tag{S2}$$

Let

$$a = \frac{a'}{a_0}, c = \frac{c'}{a_0}, x = \frac{x'}{a_0}, K_d = K_d{}'a_0, K_a = K_a{}'a_0 \tag{S3}$$

Then the kinetic equation is

$$\frac{dx}{dt} = K_d(a-x)(c-x) - K_a x^2 \tag{S4}$$

If $K_d < K_a$, analytical solution of equation (S4) gives the integrated form of the kinetic equation:

$$x = \frac{Ae^{-\sqrt{D}t}-1}{Ae^{-\sqrt{D}t}+1}\cdot\frac{\sqrt{D}}{2C}-\frac{B}{2C} \tag{S5}$$

where the parameters A, B, C and D are related to the kinetic constants (K_d, K_a) and the initial total concentration of species (a, c) *via*

$$\begin{aligned} B &= -K_d(a+c) \\ C &= K_d - K_a \\ D &= K_d{}^2(a-c)^2 + 4acK_dK_a \\ A &= \frac{B+\sqrt{D}}{\sqrt{D}-B} \end{aligned} \tag{S6}$$

For the NP dissolution reaction, $K_d < K_a$ is appropriate. Thus, the time-dependent PL intensity is described as

$$I_t = I\cdot x = I\cdot\left(\frac{Ae^{-\sqrt{D}t}-1}{Ae^{-\sqrt{D}t}+1}\cdot\frac{\sqrt{D}}{2C}-\frac{B}{2C}\right) \tag{S7}$$

where I is the proportionality constant between I_t and x. At the dissociation-association equilibrium, $e^{-\sqrt{D}t}\approx 0$. Thus, the PL intensity of NPs dissolved in the enhancer solution at the equilibrium

$$I_{eq} = I\cdot x_{eq} = I\cdot\frac{\left[\sqrt{(a-c)^2+4ac/K_{eq}}-(a+c)\right]}{2(1/K_{eq}-1)} \tag{S8}$$

where x_{eq} is the amount of dissolved NPs at equilibrium; $K_{eq} = K_d / K_a$ is the equilibrium constant. The equilibration time

$$t_{eq} \propto 1/\sqrt{D} = 1/\left[\sqrt{K_d{}^2(a-c)^2+4acK_dK_a}\right] \tag{S9}$$

According to equation (S8), the higher equilibrium constant (K_{eq}) is related to the larger amount of NPs dissolved in the enhancer solution at the dissociation-association equilibrium. Meanwhile, it can be concluded from equation (S9) that the higher dissociation / association rate constant (K_d / K_a) implies the shorter equilibration time for the dissolution reaction.

II. Supplementary Tables

Table S1. Crystallographic parameters of lutetium oxyfluoride and some typical lanthanide fluorides.

Material	Crystal system	Formula weight (g/mol)	Cell volume (A^3)	Z [a)]	Molar density of Ln^{3+} (mmol/cm^3)	Mass density (g/cm^3)	JCPDS No.
$Lu_6O_5F_8$	orthorhombic	1281.82	945.00	4	42.17	9.01	Ref. 8
$NaEuF_4$	hexagonal	250.96	115.25	1.5	21.61	5.42	049-1897
$NaLuF_4$	hexagonal	273.96	104.13	1.5	23.92	6.55	027-0726
$LiLuF_4$	tetragonal	257.91	276.92	4	23.99	6.19	027-1251

a) Formula units per cell.

Table S2. Elemental analysis of as-synthesized $Lu_6O_5F_8$:Eu (40 mol%) NPs. [a)]

Measurement	Molar Ratio [b)]	
	Lu	Eu
EDX	58.25%	41.75%
ICP-AES	61.28%	38.72%

a) The noted Ln^{3+} molar percentage was as designed in the precursor solution as compared to the total amount of Ln^{3+} ions.

b) The molar ratio is normalized so that $n(Lu^{3+}) + n(Eu^{3+}) = 100\%$.

Table S3. Parameters obtained from kinetic fitting of the time-dependent dissolution-enhanced PL signal. [a)]

pH	K_d (s^{-1})	K_a (s^{-1})	K_{eq}	I
1.00	—	—	—	—
1.91	1.45×10^{-2}	4.31×10^{-2}	3.37×10^{-1}	54467
2.30	1.58×10^{-2}	2.07×10^{-2}	7.63×10^{-1}	95135
2.76	1.67×10^{-2}	1.98×10^{-2}	8.41×10^{-1}	155644
2.92	9.52×10^{-3}	2.31×10^{-2}	4.13×10^{-1}	160547

pH	K_{d1}	K_{a1}	K_{d2}	K_{a2}	K_{eq1}	K_{eq2}	I_1	I_2
3.60	2.50×10^{-2}	3.16×10^{-2}	4.52×10^{-3}	1.00×10^{-2}	7.92×10^{-1}	4.52×10^{-1}	66685	75461
5.50	2.93×10^{-2}	3.87×10^{-2}	2.29×10^{-3}	6.15×10^{-3}	7.58×10^{-1}	3.71×10^{-1}	33763	44829

a) At pH 3.60 and 5.50, the parameters K_{di}, K_{ai}, K_{eqi} and I_i (i = 1, 2) were obtained from fitting of the time-dependent dissolution-enhanced PL signal to two independent kinetic equations (see the text description in Figure S12 for the fitting detail).

Table S4. Limit of detection for PSA based on some typical lanthanide luminescent inorganic/organic nanoprobes.

Nanoprobes	Size (nm)	Bioconjugation	Limit of detection (pg/mL)	Assay format	Ref.
Eu^{3+} chelates embedded polystyrene NPs	41±11	Antibody	1.6	TRPL	9
Tb^{3+} chelates embedded polystyrene NPs	51±9	Antibody	2.4	TRPL	9
Sm^{3+} chelates embedded polystyrene NPs	46±6	Antibody	10.1	TRPL	9
Dy^{3+} chelates embedded polystyrene NPs	46±17	Antibody	114.2	TRPL	9
Silica-coated Tb^{3+} chelates NPs	42±3	Streptavidin	7.0	TRPL	10
Eu^{3+} chelates covalently bond silica NPs	56 ±4	Streptavidin	210	TRPL	11
$NaYF_4$:Yb^{3+}/Er^{3+},Mn^{2+} nanorods	~19×42	Antibody	112.9	UC-FRET	12
$NaYF_4$:Yb^{3+}/Er^{3+} NPs	~30	Antibody	150	UC	13
**$Lu_6O_5F_8$:Eu^{3+} NPs**	**~5**	**Avidin**	**0.52**	**TRPL**	**This work**

III. Supplementary Figures

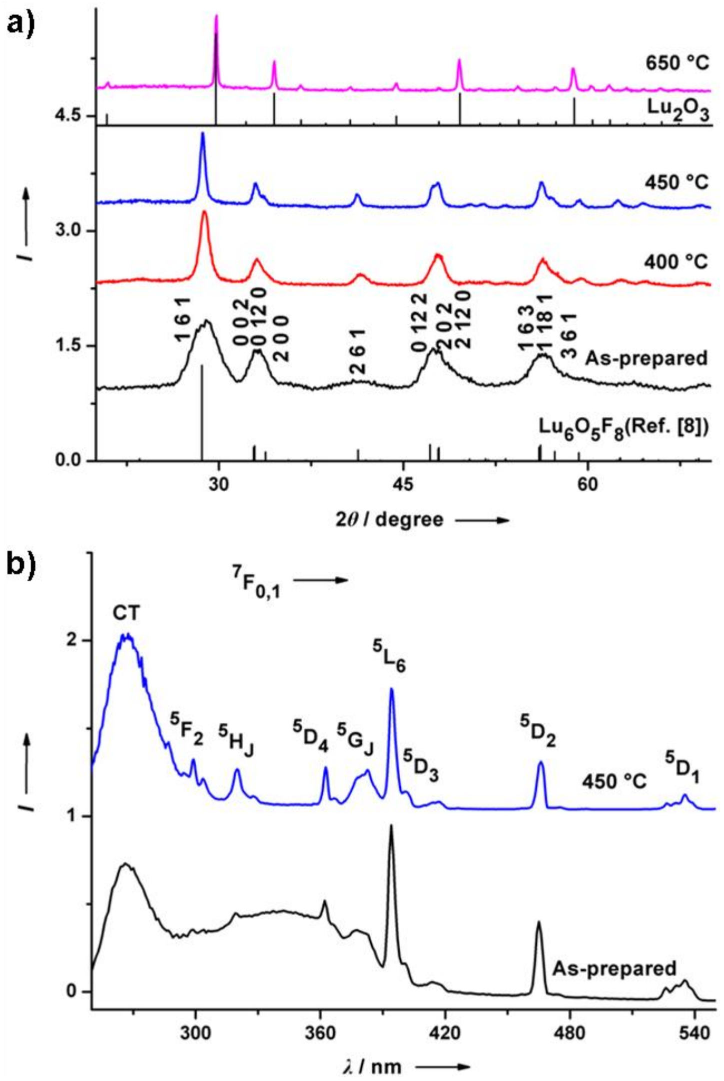

Figure S1. a) XRD patterns of as-prepared $Lu_6O_5F_8:Eu^{3+}$ (5 mol%) NPs and NPs annealed at 400, 450 and 650 ℃ for 72 h. The XRD patterns of as-prepared NPs and NPs annealed at 400, 450 ℃ match well with the standard pattern of orthorhombic-phase $Lu_6O_5F_8$ (Ref. 8). However, the XRD pattern of NPs annealed at 650 ℃ match well with cubic Lu_2O_3 (JCPDS NO. 43-1021), indicative of the thermal decomposition of $Lu_6O_5F_8$ to Lu_2O_3 at 650 ℃. b) Corresponding PL excitation spectra of $Lu_6O_5F_8:Eu^{3+}$ (5 mol%) NPs and NPs annealed at 450 ℃ by monitoring the $^5D_0 \rightarrow {}^7F_2$ transition of Eu^{3+} at 613 nm. The identical sharp PL excitation lines attributed to intra-4f transitions of Eu^{3+} were observed for the as-prepared NPs and NPs annealed at 450 ℃, which indicates that the Eu^{3+} ions experienced nearly the same crystal-field environment in as-prepared and annealed NPs, thereby substantiating the orthorhombic phase of as-prepared $Lu_6O_5F_8:Ln^{3+}$ NPs.

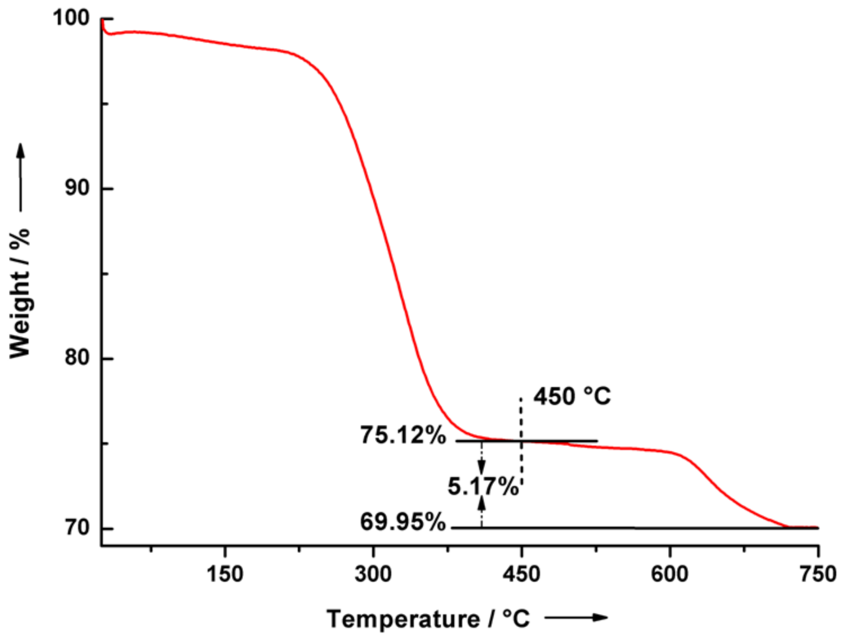

Figure S2. TGA curve of as-prepared $Lu_6O_5F_8$:Eu^{3+} (5 mol%) NPs under flowing air atmosphere in the temperature range of 25-750 ℃ at a rate of 10 ℃/min. The weight losses occurring below 410 ℃ are attributed to the removal of adsorbed moisture and the burning of organic oleate ligands on the NP surface. Between 410 ℃ and 490 ℃, the mass of the sample was stabilized, verifying the complete burning of oleate ligands on the NP surface. Above 490 ℃, a continuous weight loss ascribed to the thermolysis of $Lu_6O_5F_8$ NPs was observed, and no weight loss was observed above 720 ℃. As a result, it is concluded that the final product (*i.e.* Lu_2O_3) was obtained after the complete thermolysis of $Lu_6O_5F_8$ NPs, as confirmed by the XRD result of as-prepared NPs annealed at 650 ℃ for 72 h (Figure S1). The weight loss of 5.17% between 450 ℃ and 750 ℃, owing to the complete decomposition of $Lu_6O_5F_8$ to Lu_2O_3, is consistent with the theoretically calculated weight loss (5.19%), thus further verifying the formation of orthorhombic $Lu_6O_5F_8$ NPs.

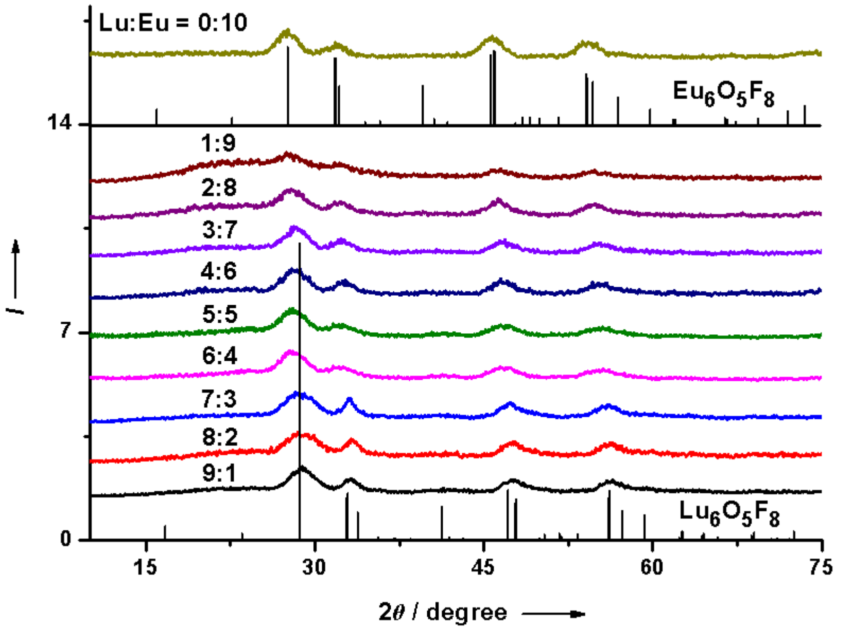

Figure S3. XRD patterns of as-prepared $Lu_6O_5F_8$:Eu^{3+} (x mol%) NPs (x = 10, 20, 30, 40, 50, 60, 70, 80, 90, and 100) with different molar ratio of Lu^{3+}:Eu^{3+} (9:1, 8:2, 7:3, 6:4, 5:5, 4:6, 3:7, 2:8, 1:9, and 0:10). The XRD peaks of as-prepared $Lu_6O_5F_8$:Eu^{3+} (100 mol%) NPs match well with the standard pattern of orthorhombic-phase $Eu_6O_5F_8$ (JCPDS No. 26-0637), which is isomorphic to orthorhombic-phase $Lu_6O_5F_8$. With the increase of Eu^{3+} doping concentration, the diffraction peaks of as-prepared NPs gradually shift to lower angles, indicating the formation of homogeneous orthorhombic $(Lu_{1-x}Eu_x)_6O_5F_8$ ($0 < x \leq 1$) NPs.

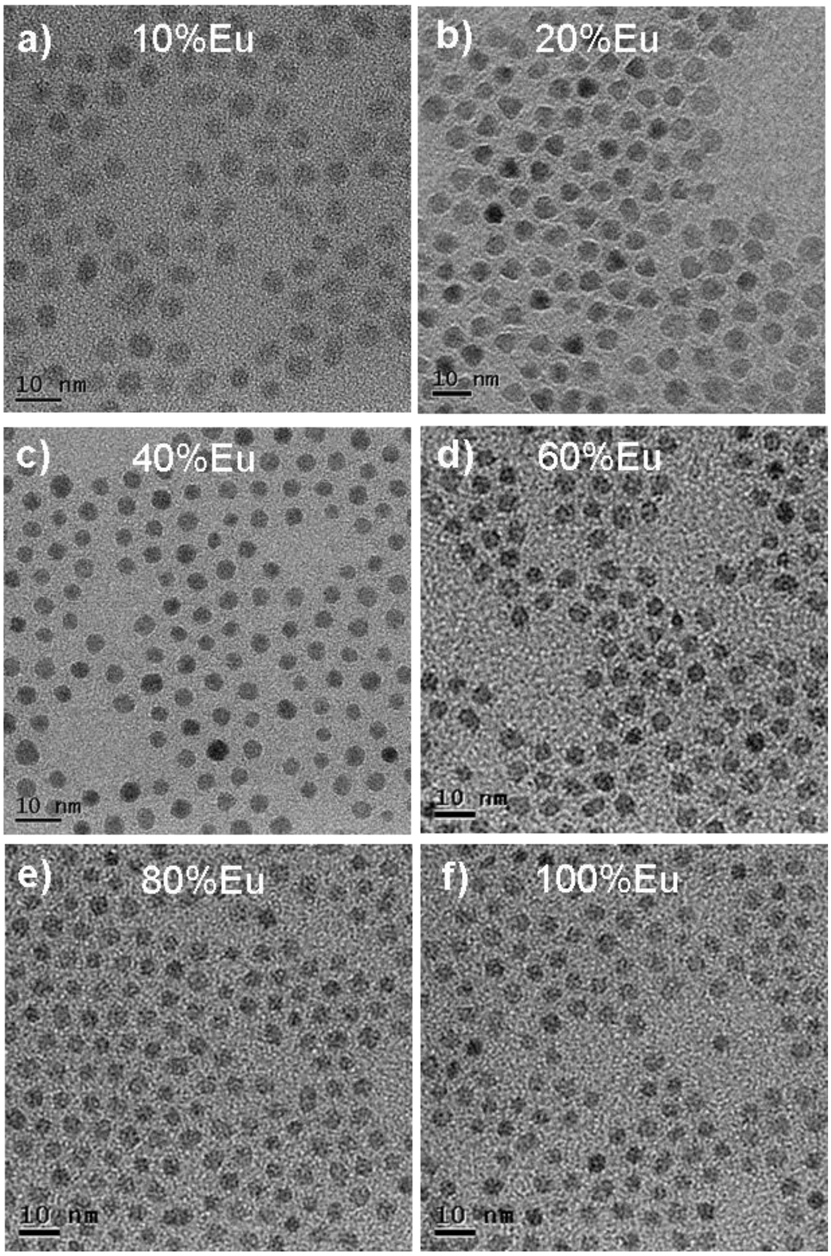

Figure S4. TEM images of as-prepared $Lu_6O_5F_8$ NPs doped with a) 10 mol% Eu^{3+}, b) 20 mol% Eu^{3+}, c) 40 mol% Eu^{3+}, d) 60 mol% Eu^{3+}, e) 80 mol% Eu^{3+}, and f) 100 mol% Eu^{3+}, respectively, which show no noticeable variation of size and morphology of NPs.

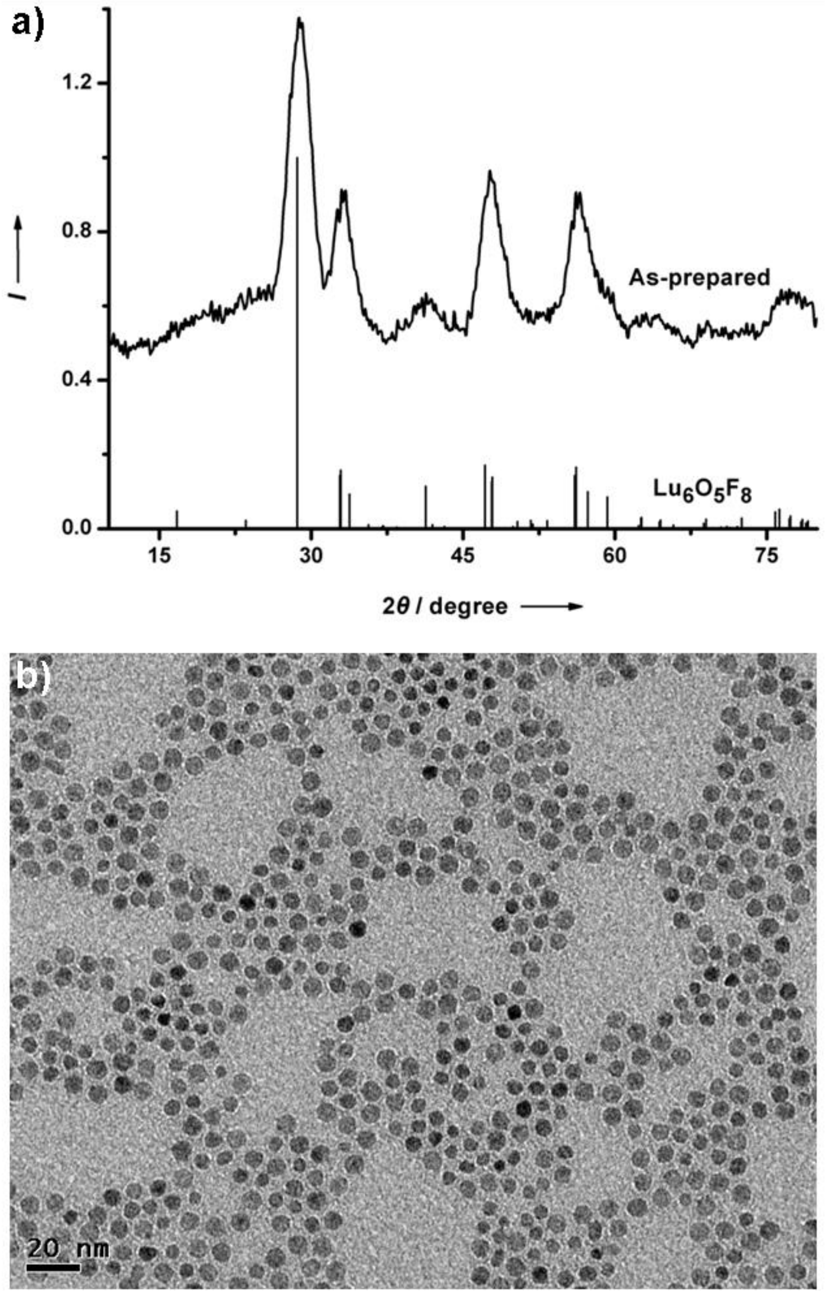

Figure S5. a) XRD pattern of as-prepared $Lu_6O_5F_8$:Yb^{3+}/Er^{3+} (10/2 mol%) NPs. All diffractions peaks match well with the standard pattern of orthorhombic $Lu_6O_5F_8$, indicating the formation of highly crystalline $Lu_6O_5F_8$ NPs. b) TEM image of as-prepared $Lu_6O_5F_8$:Yb^{3+}/Er^{3+} NPs, which shows no noticeable variation of the size and morphology of NPs when changing the doped Ln^{3+} contents.

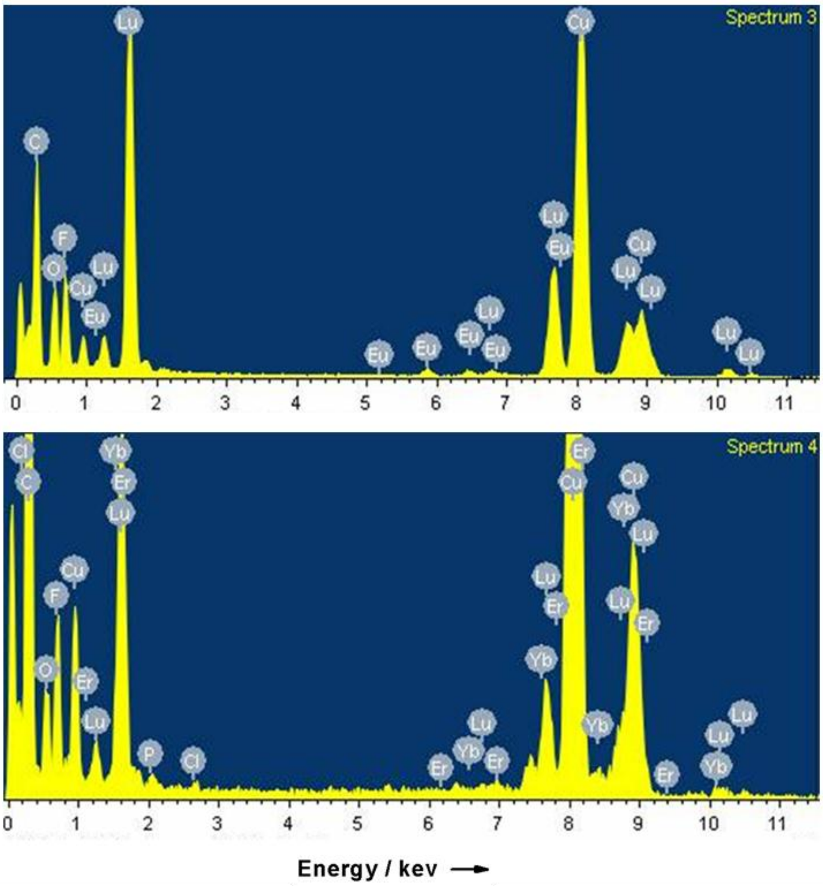

Figure S6. Energy-dispersive X-ray (EDX) spectroscopy analysis of as-prepared $Lu_6O_5F_8:Eu^{3+}$ (5 mol%) NPs (upper) and $Lu_6O_5F_8:Yb^{3+}/Er^{3+}$ (10/2 mol%) NPs (lower), revealing the presence of the doped elements of Eu or Yb/Er in $Lu_6O_5F_8$ NPs.

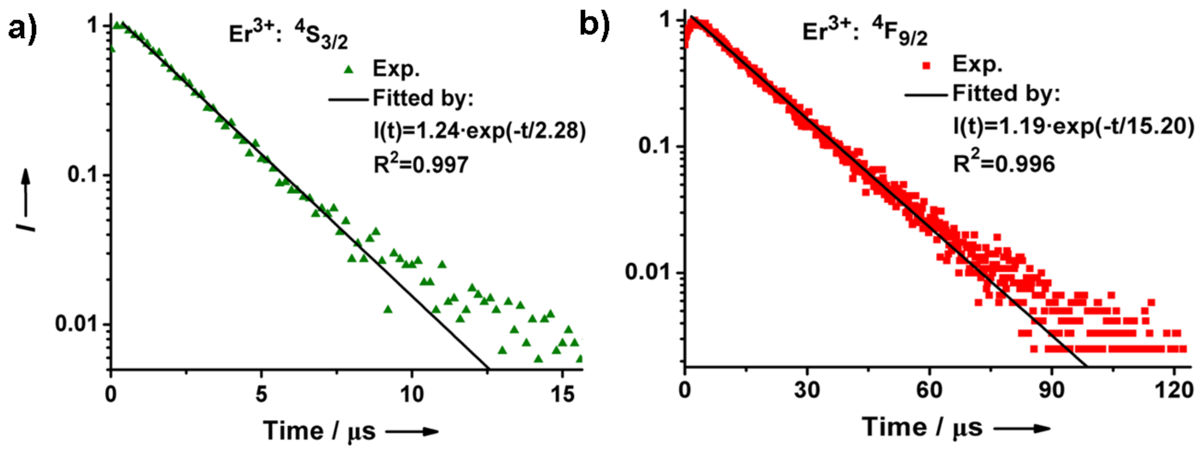

Figure S7. UCL decays from a) $^4S_{3/2}$ and b) $^4F_{9/2}$ of Er^{3+} in as-prepared $Lu_6O_5F_8$:Yb^{3+}/Er^{3+} (10/2 mol%) NPs upon excitation at 980 nm. The UCL lifetime was determined by single-exponential fit to the decay with the regression equation of $I(t)=A\ exp(-t/\tau)$, where A is pre-exponential factor; τ is UCL lifetime. Accordingly, the UCL lifetimes of $^4S_{3/2}$ and $^4F_{9/2}$ were determined to be 2.3 and 15.2 μs, respectively.

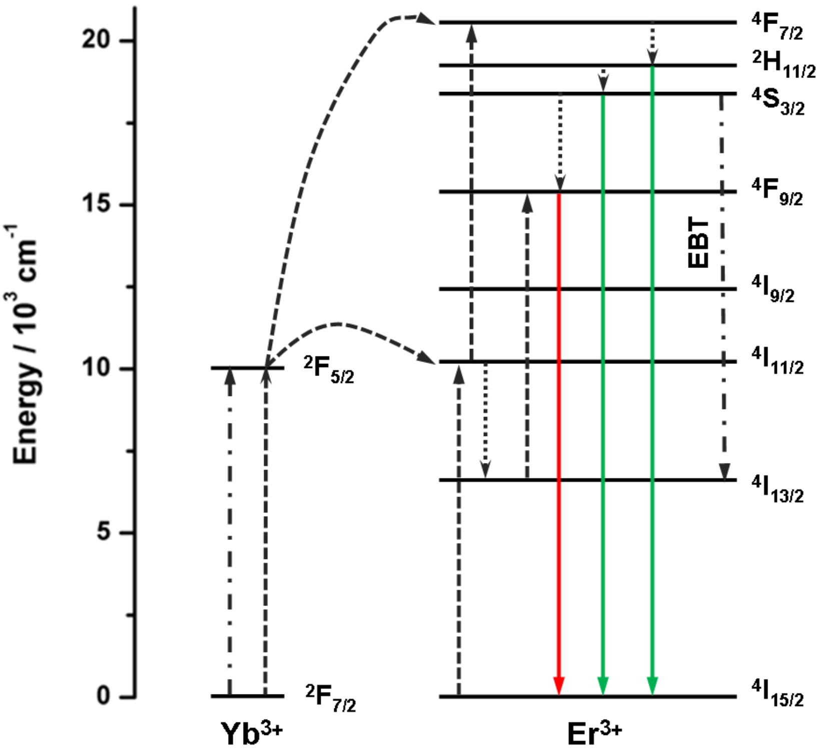

Figure S8. Energy level diagrams of Er^{3+} and Yb^{3+} ions and the proposed UCL mechanism for the large red-to-green ratio emission in $Lu_6O_5F_8$:Yb^{3+}/Er^{3+} (10/2 mol%) NPs. The dashed, dotted, dash-dotted and solid arrows represent the excitation, nonradiative relaxation, energy back transfer (EBT) $^4S_{3/2}(Er^{3+})$ + $^2F_{7/2}(Yb^{3+}) \rightarrow {}^4I_{13/2}(Er^{3+})$ + $^2F_{5/2}(Yb^{3+})$, and radiative transition processes, respectively.

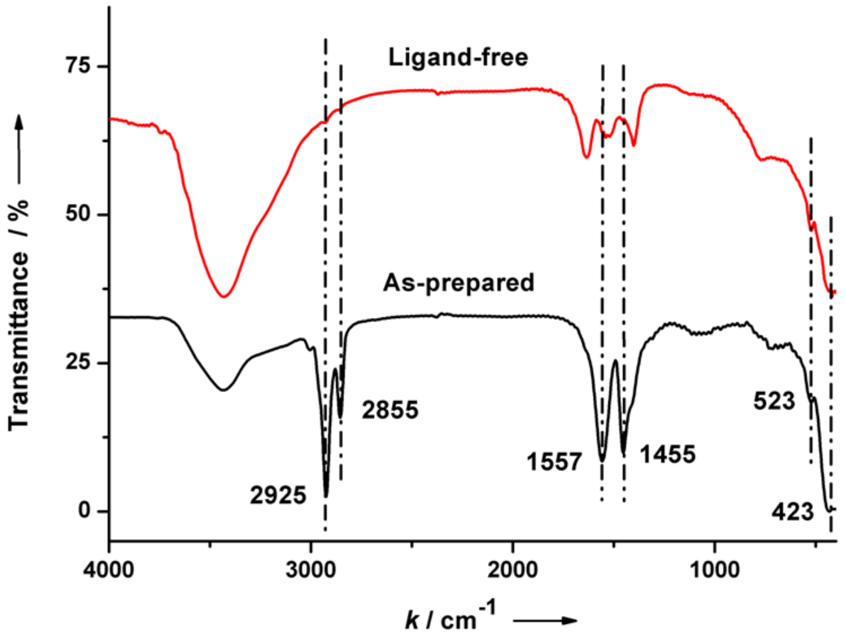

Figure S9. Comparison of FTIR spectra for as-prepared and ligand-free $Lu_6O_5F_8:Eu^{3+}$ (40 mol%) NPs. The original asymmetric and symmetric stretching vibrations of methylene (-CH_2-) in the long alkyl chain peaking at 2925 and 2855 cm^{-1}, and the asymmetric and symmetric stretching vibrations of the carboxylic group (-COO^-) peaking at 1557 and 1455 cm^{-1} disappeared in ligand-free NPs. Besides, the strong band centered at 423 cm^{-1} and the relatively weak band at 523 cm^{-1}, which are attributed to vibrational modes of the lutetium oxyfluoride lattice,[14] remained essentially unchanged in ligand-free NPs. These features corroborate the successful removal of oleate ligands from the surface of NPs.

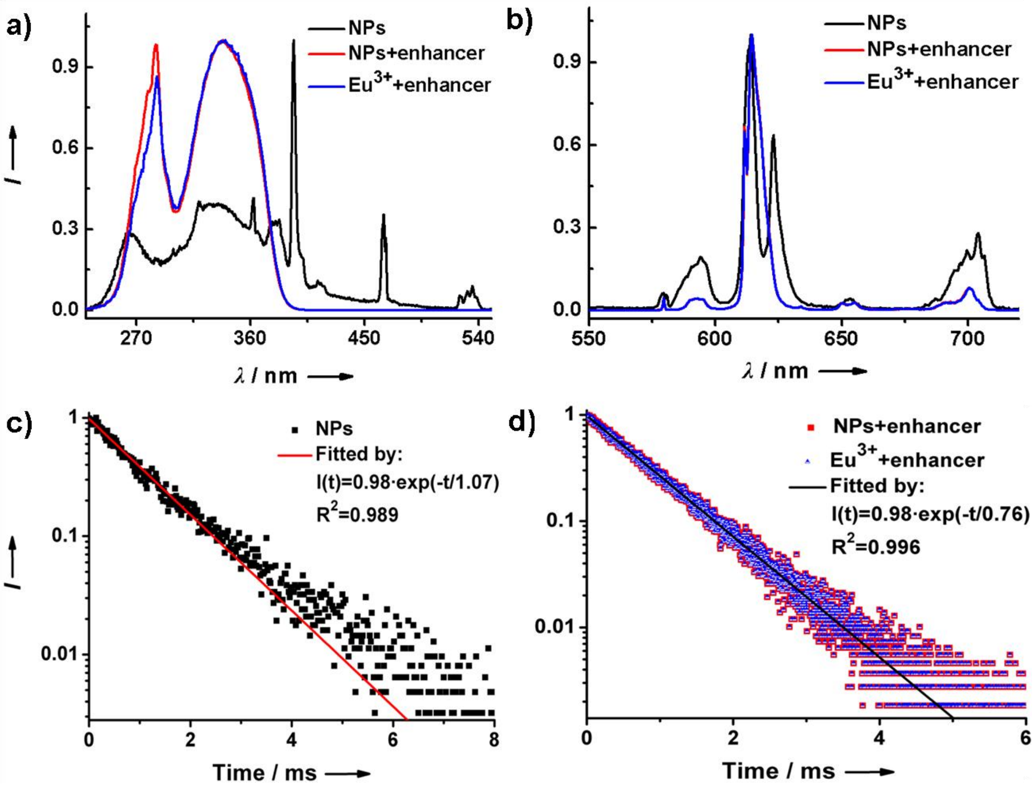

Figure S10. Normalized PL a) excitation spectra and b) emission spectra of ligand-free $Lu_6O_5F_8:Eu^{3+}$ (40 mol%) NPs in buffer solution and the enhancer solution, and Eu^{3+} in the enhancer solution (i.e. β-NTA-Eu^{3+}-TOPO ternary complex), respectively. c,d) Corresponding PL decays from $^5D_0$ by monitoring the Eu^{3+} emission at 614 nm for c) ligand-free NPs in buffer solution, and d) ligand-free NPs in the enhancer solution and free Eu^{3+} ions in the enhancer solution, respectively.

In a) and b), the PL excitation and emission spectra of the NPs in buffer solution (5 mg/mL, pH 2.76) exhibited characteristic and sharp spectral peaks with dominant excitation and emission bands centered at 394 nm and 614 nm, respectively. By contrast, the NPs dissolved in the enhancer solution (0.5 μg/mL, pH 2.76) exhibited an unusual broad band at approximately 340 nm in the excitation spectrum and distinct PL branching ratios for the $^5D_0 \rightarrow {}^7F_{0-4}$ transitions of Eu^{3+} in the emission spectrum. These bands were found to be identical to those of β-NTA-Eu^{3+}-TOPO ternary complex formed by dissolving $Eu(CF_3COO)_3$ in the enhancer solution (1 μg/mL). In c) and d), the PL lifetime was determined by single-exponential fit to the decay with the regression equation of $I(t)=A\ \exp(-t/\tau)$, where A is pre-exponential factor; τ is PL lifetime. The PL lifetime of the NPs in buffer solution was determined to be 1.07 ms, which is much longer than the PL lifetime (0.76 ms) observed in the NPs dissolved in the enhancer solution and the β-NTA-Eu^{3+}-TOPO ternary complex. These results show unambiguously that the enhanced Eu^{3+} PL of the NPs dissolved in the enhancer solution originates from the formed β-NTA-Eu^{3+}-TOPO ternary complex instead of the NPs themselves.

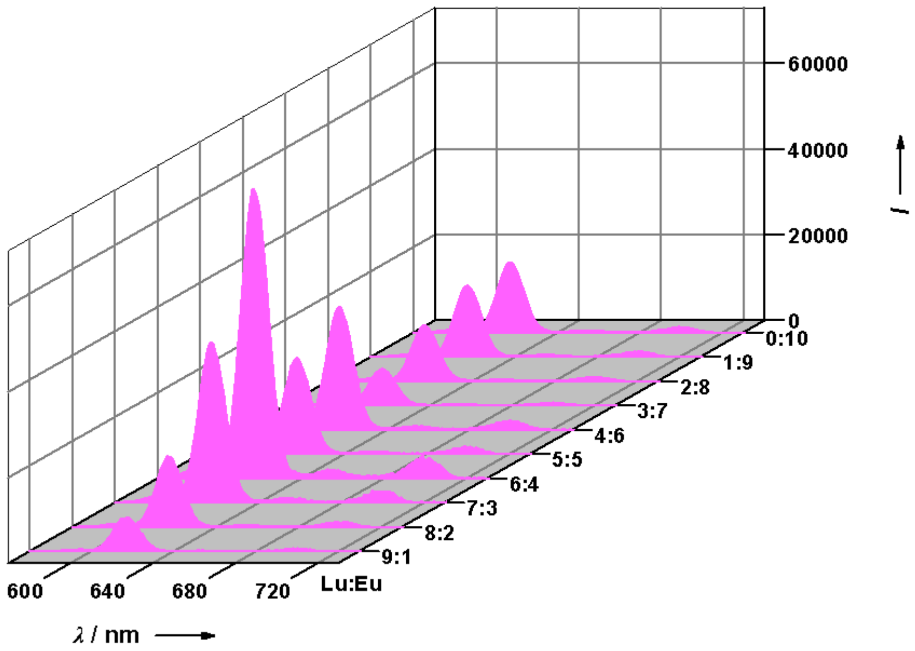

Figure S11. Comparison of dissolution-enhanced PL spectra upon UV excitation at 340 nm for ligand-free $Lu_6O_5F_8:Eu^{3+}$ (x mol%) NPs (x = 10, 20, 30, 40, 50, 60, 70, 80, 90, and 100) with different molar ratio of $Lu^{3+}:Eu^{3+}$ (9:1, 8:2, 7:3, 6:4, 5:5, 4:6, 3:7, 2:8, 1:9, and 0:10). The dissolution-enhanced PL signal of ligand-free $Lu_6O_5F_8:Eu^{3+}$ (x mol%) NPs (0.026 μg/mL in buffer solution at pH 2.76, 100 μL) was recorded at RT after 10 min upon addition of the enhancer solution (pH 2.76, 100 μL). Specifically, when the molar ratio of $Lu^{3+}:Eu^{3+}$ is 6:4, the PL intensity is the strongest.

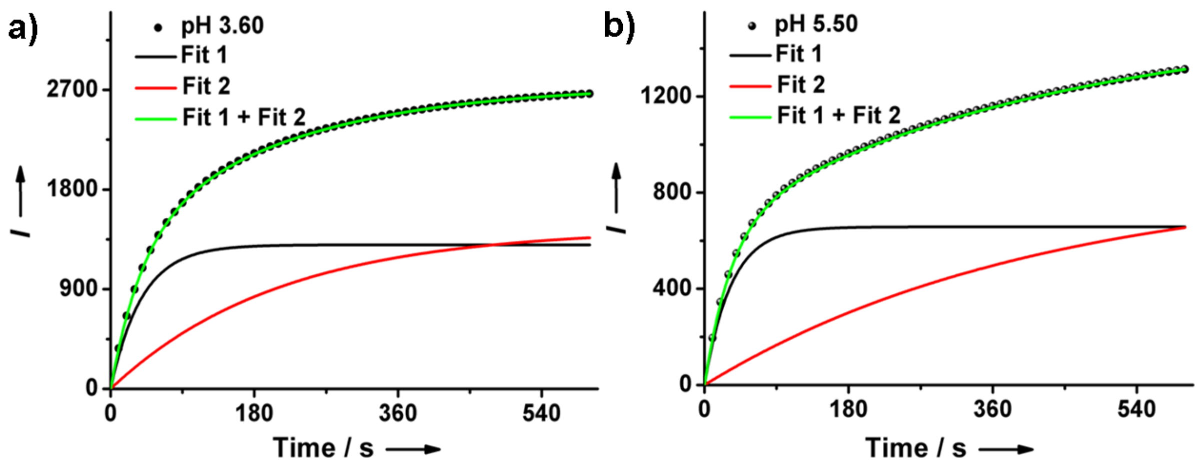

Figure S12. Kinetic fit to time-dependent dissolution-enhanced PL signal from ligand-free $Lu_6O_5F_8:Eu^{3+}$ (40 mol%) NPs (50 μg/mL) dissolved in the enhancer solution at pH 3.60 and 5.50. At pH 3.60 and 5.50, the kinetic equation S7 did not fit the time-dependent dissolution-enhanced PL signal very well. Alternatively, the dissolution reaction of NPs in the enhancer solution at pH 3.60 or 5.50 can be well described by two independent dissolution reactions, namely,

$$I_t = I_1 \cdot x = I_1 \cdot \left(\frac{A_1 e^{-\sqrt{D_1} t} - 1}{A_1 e^{-\sqrt{D_1} t} + 1} \cdot \frac{\sqrt{D_1}}{2C_1} - \frac{B_1}{2C_1} \right) + I_2 \cdot \left(\frac{A_2 e^{-\sqrt{D_2} t} - 1}{A_2 e^{-\sqrt{D_2} t} + 1} \cdot \frac{\sqrt{D_2}}{2C_2} - \frac{B_2}{2C_2} \right)$$

where the parameters A_i (i = 1, 2), B_i, C_i and D_i are related to the kinetic constants (K_{di}, K_{ai}) and the initial total concentration of species (a, c) *via*

$$B_i = -K_{di}(a+c)$$
$$C_i = K_{di} - K_{ai}$$
$$D_i = K_{di}{}^2(a-c)^2 + 4acK_{di}K_{ai}$$
$$A_i = \frac{B_i + \sqrt{D_i}}{\sqrt{D_i} - B_i}$$

Different from the case of NP dissolution at pH 2.92 or lower, the time-dependent dissolution-enhanced PL signal of NPs dissolved in the enhancer solution at pH 3.60 or 5.50 was well fitted by two independent kinetic equations (Fit 1 and Fit 2), which represent the rapid and slow dissolution reaction of NPs, respectively. Such a phenomenon is due possibly to the nonuniform distribution of H^+ concentration in the enhancer solution at pH 3.60 or 5.50 caused by the produced weak acid (HF) during the dissolution reaction of NPs.

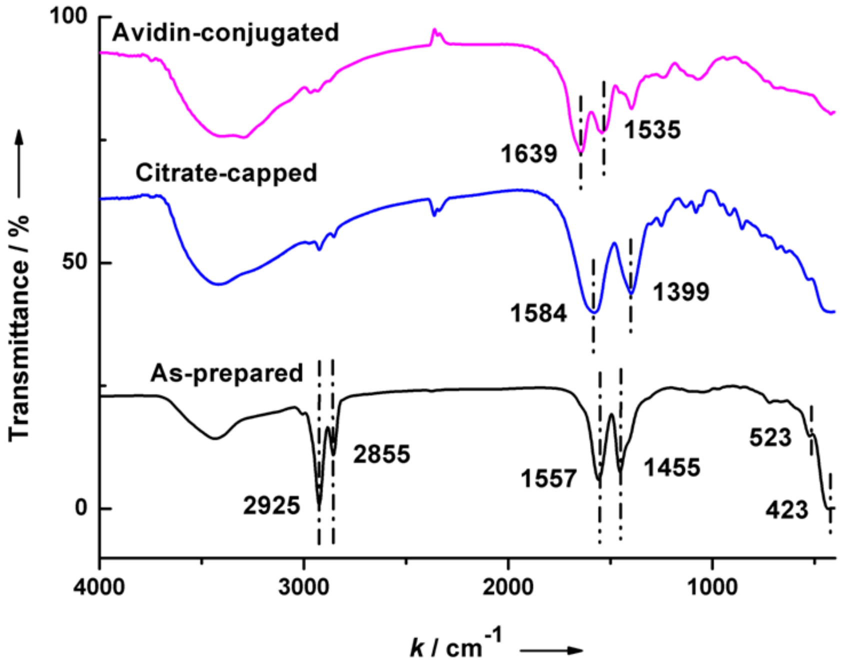

Figure S13. Comparison of FTIR spectra for as-prepared, citrate-capped and avidin-conjugated $Lu_6O_5F_8:Eu^{3+}$ (40 mol%) NPs. The bands peaking at approximately 2925 and 2855 cm^{-1} in as-prepared NPs, which can be assigned to the asymmetric and symmetric stretching vibrations of methylene ($-CH_2-$) in the long alkyl chain, disappeared in citrate-capped NPs. In addition, bands centered at 1557 and 1455 cm^{-1} are associated with the asymmetric and symmetric stretching vibrations of the carboxylic group ($-COO^-$) of the bound oleic acid, and in the case of citrate-capped NPs, these bands were shifted to 1584 and 1399 cm^{-1}, respectively. These features indicate the successful ligand exchange of as-prepared NPs with citrate. Strong bands at 1639 and 1535 cm^{-1}, associated with the amide bands I and II of avidin, were observed in avidin-conjugated NPs, thus verifying the conjugation of avidin on the surface of citrate-capped NPs.

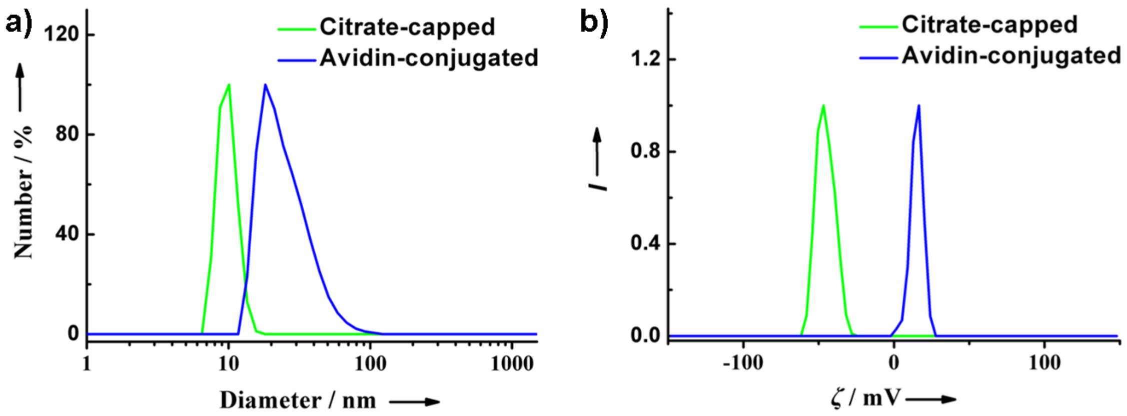

Figure S14. a) Hydrodynamic diameter (HD) distribution and b) ζ-potential of citrate-capped and avidin-conjugated $Lu_6O_5F_8:Eu^{3+}$ (40 mol%) NPs obtained from the DLS measurement. In a), citrate-capped NPs had a HD of 9.8 nm, whereas avidin-conjugated NPs showed a larger HD of 18.4 nm. Meanwhile, in b), after avidin conjugation, the ζ-potential for NPs dispersed in aqueous solution (pH 6.90) changed from -46.9 mV to 15.8 mV, as a result of the positively-charged avidin bound on the NP surface.

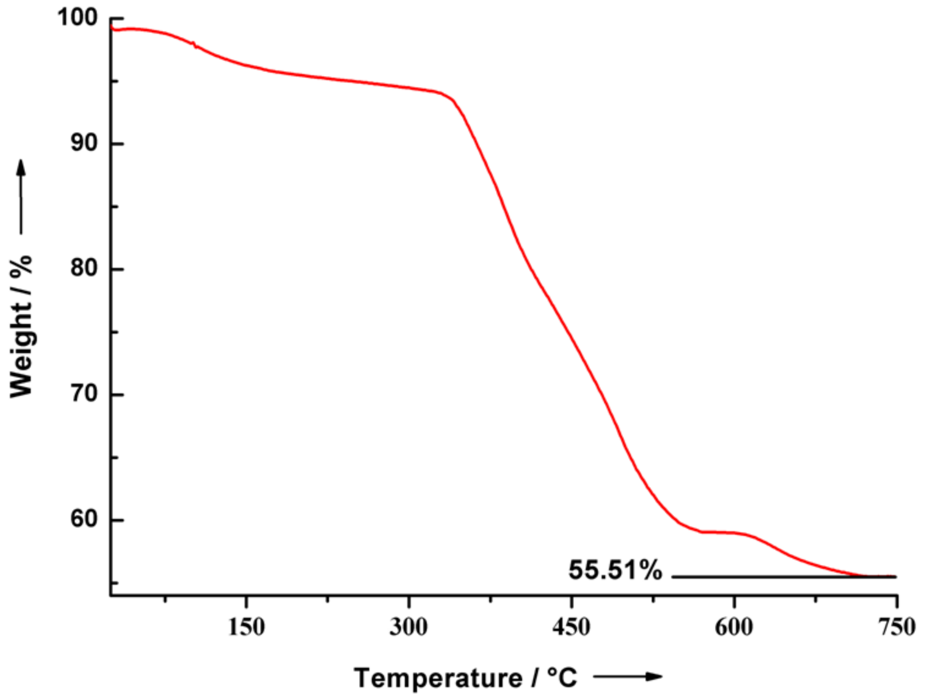

Figure S15. TGA curve of avidin-conjugated $Lu_6O_5F_8:Eu^{3+}$ (40 mol%) NPs under flowing air atmosphere in the temperature range of 25-750 ℃ at a rate of 10 ℃/min. The different weight losses occurring below 720 ℃ are attributed to the removal of adsorbed moisture, the burning of organic citrate ligands and conjugated avidin on the NP surface, and the thermal decomposition of $Lu_6O_5F_8$ to Lu_2O_3, respectively. Based on the weight percentage of Lu_2O_3 at 750 ℃ (55.51%), the weight percentage of $Lu_6O_5F_8$ in avidin-conjugated $Lu_6O_5F_8$ NPs can be calculated to be 59.80%.

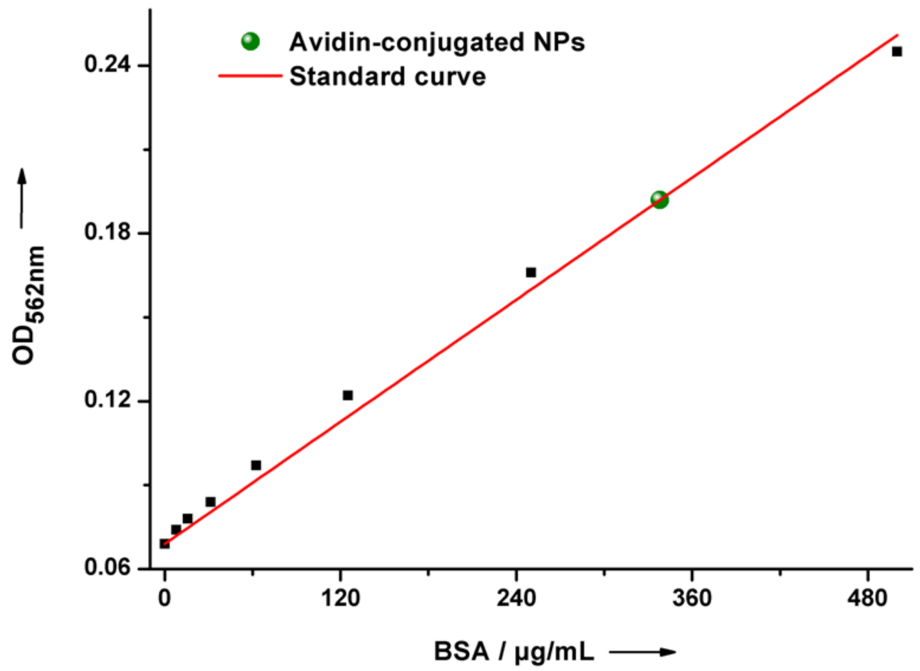

Figure S16. Quantitative analysis of avidin in avidin-conjugated $Lu_6O_5F_8:Eu^{3+}$ (40 mol%) NPs. The amount of avidin bound to the surface of citrate-capped NPs was roughly estimated to be 3.31×10^{-6} mol/g using commercial BCA protein assay kit and BSA as a reference. Furthermore, based on the above quantitative value of avidin, and the calculated weight percentage of $Lu_6O_5F_8$ (59.80%) in avidin-conjugated $Lu_6O_5F_8$ NPs derived from Figure S15, the number of avidin ($n_{\mathrm{Avidin/\ Lu6O5F8}}$) conjugated to each NP can be calculated by the following equation to be ~1.8.

$$n_{Avidin/Lu6O5F8} = \frac{3.31\times10^{-6}\times N_{\mathrm{A}}}{Weight\%/(\rho\times1/6\pi d^{3})}\times10^{-21}$$

where N_A is the Avogadro's number; *Weight%* is the weight percentage of $Lu_6O_5F_8$, which is 59.80%; ρ is the mass density of $Lu_6O_5F_8:Eu^{3+}$ (40 mol%), which is 8.26 g/cm^3; d is the average diameter of $Lu_6O_5F_8:Eu^{3+}$ NP, which is ~5 nm.

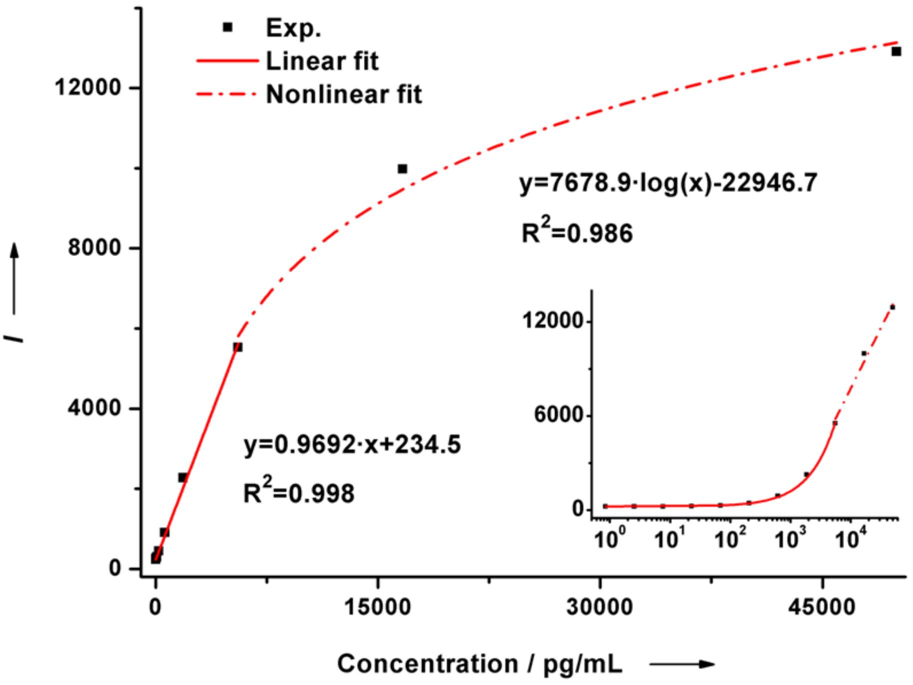

Figure S17. Regression analysis of the calibration curve plotted in the linear scale for the PSA assay based on $Lu_6O_5F_8:Eu^{3+}$ (40 mol%) NPs. Inset: the regression analysis of the calibration curve displayed in the semi-logarithmic scale. In the range of 8.5×10^{-4}-50.0 ng/mL, the piecewise fit to the calibration curve was applied: in the range of 8.5×10^{-4}-5.6 ng/mL, the linearity in the calibration curve was observed with the regression equation of y=0.9692 ·x+234.5 (R^2=0.998); in the range of 5.6-50.0 ng/mL, the curve was fitted with the regression equation of y=7678.9 ·log(x)-22946.7 (R^2=0.986).

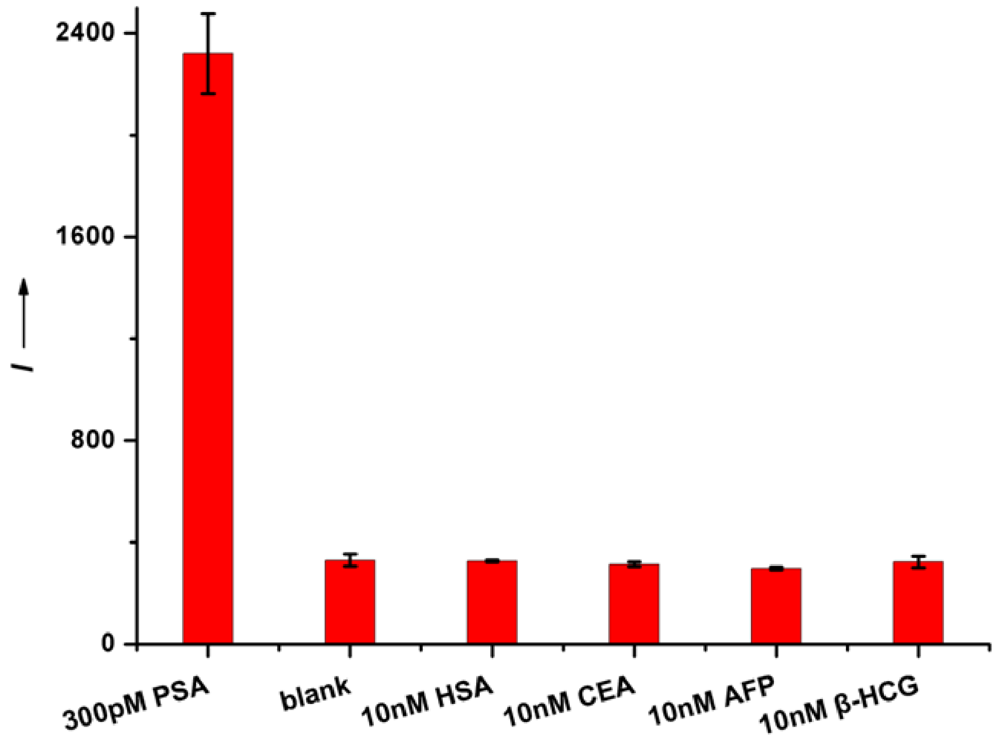

Figure S18. Specificity of the PSA assay in complex serum samples. The prepared serum samples from a healthy male with an original PSA level of 0.54 ng/mL (15.88 pM) were added with 300 pM PSA or 10 nM other proteins (*e.g.*, HSA/CEA/AFP/β-HCG). In the blank control, no targets were added. The PL intensities of all samples were measured under the same conditions. Each data point represents the mean (±standard deviation) of triplicate experiments.

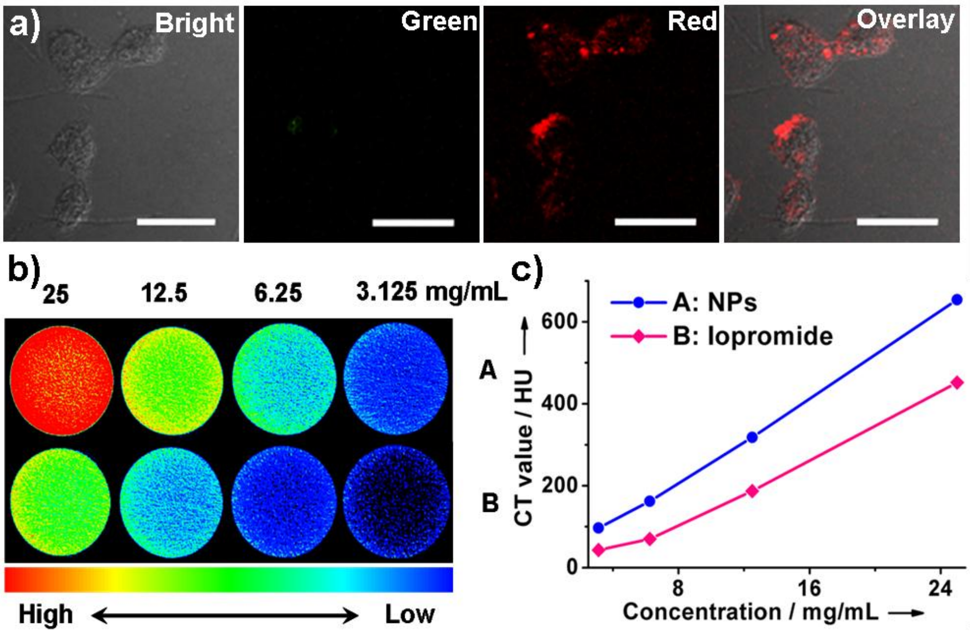

Figure S19. a) Confocal laser scanning microscopy images of H1299 cells after incubation with citrate-capped $Lu_6O_5F_8:Yb^{3+}/Er^{3+}$ NPs. Panel 1-3 show the bright-field, green and red UCL images, respectively. Panel 4 is the overlay image of Panel 1-3 (scale bar = 30 μm). b) Color-mapped CT images of cross-section of the centrifuge tubes with $Lu_6O_5F_8:Yb^{3+}/Er^{3+}$ NPs (upper) and Iopromide (lower) in aqueous solution at different concentrations. c) CT value of $Lu_6O_5F_8:Yb^{3+}/Er^{3+}$ NPs and Iopromide in aqueous solution at different concentrations.

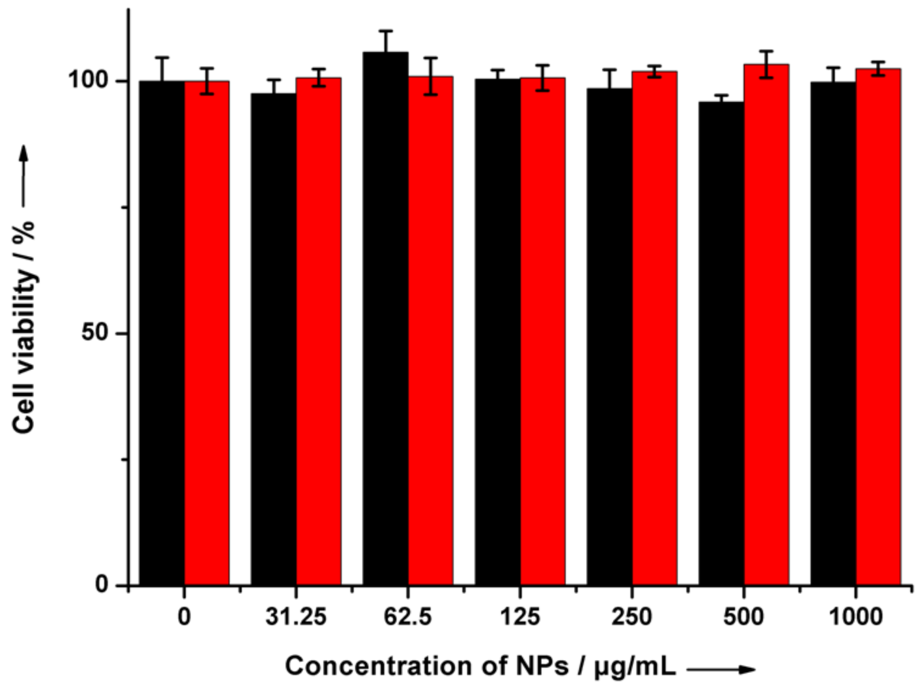

Figure S20. *In vitro* dark cytotoxicity (black) and phototoxicity (red) of citrate-capped $Lu_6O_5F_8$:Yb^{3+}/Er^{3+} NPs against HELF cells after incubation for 12 h by using MTT assay. The cell viability was determined to be larger than 95% even at a concentration as high as 1 mg/mL for NPs either in the dark or upon 980 nm light irradiation for 2 min and thus proved insignificant cytotoxicity of $Lu_6O_5F_8$:Yb^{3+}/Er^{3+} NPs.

IV. Supplementary Movie

Movie S1. The process of PSA assay based on dissolution-enhanced PL of $Lu_6O_5F_8:Eu^{3+}$ nanoprobes.

References

1. X. Sun, Y. W. Zhang, Y. P. Du, Z. G. Yan, R. Si, L. P. You and C. H. Yan, *Chem. Eur. J.*, 2007, **13**, 2320-2332.
2. J. E. Roberts, *J. Am. Chem. Soc.*, 1961, **83**, 1087.
3. N. Bogdan, F. Vetrone, G. A. Ozin and J. A. Capobianco, *Nano Lett.*, 2011, **11**, 835-840.
4. T. Y. Cao, T. S. Yang, Y. Gao, Y. Yang, H. Hu and F. Y. Li, *Inorg. Chem. Commun.*, 2010, **13**, 392-394.
5. M. L. Deng and L. Y. Wang, *Nano Res.*, 2014, **7**, 782-793.
6. F. Meiser, C. Cortez and F. Caruso, *Angew. Chem. Int. Ed.*, 2004, **43**, 5954-5957.
7. I. Hemmilä, S. Dakubu, V. M. Mukkala, H. Siitari and T. Lövgren, *Anal. Biochem.*, 1984, **137**, 335-343.
8. L. N. Guo, Y. H. Wang, Y. Z. Wang, J. Zhang, P. Y. Dong and W. Zeng, *Nanoscale*, 2013, **5**, 2491-2504.
9. P. Huhtinen, M. Kivelä, O. Kuronen, V. Hagren, H. Takalo, H. Tenhu, T. Lövgren and H. Härmä, *Anal. Chem.*, 2005, **77**, 2643-2648.
10. Z. Q. Ye, M. Q. Tan, G. L. Wang and J. L. Yuan, *Anal. Chem.*, 2004, **76**, 513-518.
11. H. F. Jiang, G. L. Wang, W. Z. Zhang, X. Y. Liu, Z. Q. Ye, D. Y. Jin, J. L. Yuan and Z. G. Liu, *J. Fluoresc.*, 2010, **20**, 321-328.
12. J. G. Zhang, S. Z. Wang, N. Gao, D. X. Feng, L. Wang and H. Q. Chen, *Biosens. Bioelectron.*, 2015, **72**, 282-287.
13. H. Päkkilä, M. Ylihärsilä, S. Lahtinen, L. Hattara, N. Salminen, R. Arppe, M. Lastusaari, P. Saviranta and T. Soukka, *Anal. Chem.*, 2012, **84**, 8628-8634.
14. T. Passuello, F. Piccinelli, M. Trevisani, M. Giarola, G. Mariotto, L. Marciniak, D. Hreniak, M. Guzik, M. Fasoli, A. Vedda, V. Jary, M. Nikl, V. Causin, M. Bettinelli and A. Speghini, *J. Mater. Chem.*, 2012, **22**, 10639-10649.
